# Nuclear Glycoprotein A Repetitions Predominant (GARP) Is a Common Trait of Glioblastoma Stem-like Cells and Correlates with Poor Survival in Glioblastoma Patients

**DOI:** 10.3390/cancers15245711

**Published:** 2023-12-05

**Authors:** Niklas Zimmer, Emily R. Trzeciak, Andreas Müller, Philipp Licht, Bettina Sprang, Petra Leukel, Volker Mailänder, Clemens Sommer, Florian Ringel, Jochen Tuettenberg, Ella Kim, Andrea Tuettenberg

**Affiliations:** 1 Department of Dermatology, University Medical Center Mainz, 55131 Mainz, Germanyplicht@uni-mainz.de (P.L.);; 2 Department of Neurosurgery, University Medical Center Mainz, 55131 Mainz, Germany; 3 Laboratory of Experimental Neurooncology, University Medical Center Mainz, 55131 Mainz, Germany; 4 Institute of Neuropathology, University Medical Center Mainz, 55131 Mainz, Germany; 5 Research Center for Immunotherapy, University Medical Center Mainz, 55131 Mainz, Germany; 6 Department of Neurosurgery, SHG-Klinikum Idar-Oberstein, 55743 Idar-Oberstein, Germany; j.tuettenberg@io.shg-kliniken.de

**Keywords:** GARP, nuclear GARP, GARP^NU+^, LRRC32, glioblastoma, glioblastoma stem-like cells

## Abstract

**Simple Summary:**

Glioblastoma (GB) is the most common primary brain tumor in adults, but it remains incurable due to its high degree of therapy resistance. Glioblastoma stem-like cells (GSCs) are believed to drive the initiation, progression, and therapy resistance of GB, making them an ideal therapeutic target to improve patient outcomes. However, due to their heterogeneity, there are no universal markers to identify GSCs. We evaluated GARP as a novel marker for GSCs and found that GARP is more stably and uniformly expressed by human GSCs, across cellular states and disease stages, than the commonly used GSC marker, CD133. Additionally, we showed that GARP is intranuclearly localized in GSCs, and we are the first to show that nuclear GARP levels (GARP^NU+^) are associated with poor patient survival. Our findings indicate that GARP/GARP^NU+^ expression is an improved marker for GSCs and suggest a potential application of GARP as a prognostic biomarker for GB.

**Abstract:**

Glioblastoma (GB) is notoriously resistant to therapy. GB genesis and progression are driven by glioblastoma stem-like cells (GSCs). One goal for improving treatment efficacy and patient outcomes is targeting GSCs. Currently, there are no universal markers for GSCs. Glycoprotein A repetitions predominant (GARP), an anti-inflammatory protein expressed by activated regulatory T cells, was identified as a possible marker for GSCs. This study evaluated GARP for the detection of human GSCs utilizing a multidimensional experimental design that replicated several features of GB: (1) intratumoral heterogeneity, (2) cellular hierarchy (GSCs with varied degrees of self-renewal and differentiation), and (3) longitudinal GSC evolution during GB recurrence (GSCs from patient-matched newly diagnosed and recurrent GB). Our results indicate that GARP is expressed by GSCs across various cellular states and disease stages. GSCs with an increased GARP expression had reduced self-renewal but no alterations in proliferative capacity or differentiation commitment. Rather, GARP correlated inversely with the expression of GFAP and PDGFR-α, markers of astrocyte or oligodendrocyte differentiation. GARP had an abnormal nuclear localization (GARP^NU+^) in GSCs and was negatively associated with patient survival. The uniformity of GARP/GARP^NU+^ expression across different types of GSCs suggests a potential use of GARP as a marker to identify GSCs.

## 1. Introduction

Glioblastoma (GB) is one of the most aggressive tumors, with an overall survival rate of approximately 15 months [1,2]. The current standard therapy consists of surgical removal of the primary tumor, radiation, and treatment with the chemotherapeutic agent, temozolomide (TMZ) [3]. A high degree of tumor infiltration into the surrounding tissue is a characteristic feature of GB, limiting the clinical efficacy of neurosurgical resection. Despite multi-modal therapy, GB recurrence after initial treatment is almost inevitable [4,5,6]. Besides the immunosuppressive properties of tumor cells, which suppress anti-tumor immune responses through microglial cells or regulatory T cells, poor prognosis is also attributed to a high degree of therapeutic resistance, either inherent or acquired by therapy, and the extraordinary intratumoral heterogeneity of GB, manifesting via the diversity of molecular and cellular subtypes/cellular states associated with GSCs [7,8,9,10].

The notorious therapeutic resistance of GB has been attributed to glioblastoma stem-like cells (GSCs), which comprise a subset of tumor cells that possess some fundamental properties of cancer stem cells, including unlimited self-renewal, aberrant differentiation response, and inherent plasticity. These characteristics enable GSCs to undergo reversible transitions between distinct cellular states in response to environmental signals [11,12].

These unique properties render GSCs capable of adapting to and surviving cytotoxic treatments that are otherwise lethal to non-stem glioma cells, thereby endowing them with the potential to reconstitute the tumor during or after therapy. GSCs are currently considered the main determinants of therapy resistance and drivers of tumor recurrence in GB. Therefore, they are arguably the most clinically relevant cellular target in gliomas. Assessments of GSCs in tumor specimens face several methodological challenges. These include: (I) the relatively low percentage of GSCs compared to the rest of the tumor cells, which are thought to be comprised primarily of non-stem glioma cells or differentiated progenies of GSCs [13,14], (II) an inhomogeneous distribution of GSCs within the tumor, which are located in specialized niches that provide a proper environment for maintaining their undifferentiated state [14], and (III) the phenotypic diversity and inherent high plasticity of GSCs, enabling dynamic transitions between different cell states accompanied by morphological alterations and changes in their phenotypic make-up [15,16]. Furthermore, GSCs possess a high degree of plasticity, which renders them capable of switching between different cellular states and distinct morphological phenotypes. Lack of definitive markers that are stably expressed on GSCs poses a further challenge to the diagnostic stratification of GB based on the evaluation of GSC content in tumor specimens [17].

Although a range of molecules like CD15, L1CAM, SOX2, and Prominin1/CD133 have been implicated as identification markers of GSCs, their diagnostic utility has been limited due to the phenotypic heterogeneity within the GSC compartment, constituted by cells in hierarchically distinct states [18,19,20,21,22,23]. For example, expression of Prominin1/CD133, historically one of the most investigated and arguably the best validated GSC marker, is sample specific, being restricted to only a subset of GSCs [24,25,26,27], and fluctuates significantly during cell cycle [28]. Furthermore, a reversible loss of CD133 expression in CD133^+^ GSCs was shown to accompany tumor propagation, as revealed in an experimental in vivo model of GB [24]. Considering that the tumor-propagating capacity of CD133^−^ GSCs is comparable to that of CD133^+^ GSCs [24], the diagnostic utility of CD133 remains uncertain [29]. Phenotypic diversity and plasticity of GSCs as a means of adaptation to the tumor microenvironment have important implications for the continuing search for GSCs markers that would be universally applicable for different subsets of GSCs and would be expressed unambiguously, regardless of cellular state.

In this regard, Glycoprotein A repetitions predominant (GARP) has recently emerged as a potential marker of human GSCs [7,30]. GARP is a type I transmembrane protein normally expressed on the surface of activated regulatory T cells, where it mediates tolerogenic functions in the tumor microenvironment of GB [7]. GARP consists of 662 amino acids and is composed of an extracellular domain with 20 leucine rich repeats, a hydrophobic transmembrane domain, and a 15 amino acid intracellular part. Recently, we have found that GARP is also expressed by different types of GB cells, including GSCs, where it shows an atypical pattern of subcellular distribution characterized by GARP localization on both the cell surface and within the nucleus (GARP^NU+^) [7]. Up until now, GARP expression in GSCs has only been shown in vitro, with several open-ended questions remaining. Namely, is GARP/GARP^NU+^ expression associated with a particular cellular state (self-renewal or differentiation) or a particular subtype of GSCs? Does the associated expression of GARP/GARP^NU+^ in GSCs persist during GB progression after therapy?

In the present study, these questions were addressed in vitro and in vivo by analyzing the expression of GARP/GARP^NU+^ in different subtypes of patient-derived GSCs with consideration of intratumoral heterogeneity and the longitudinal changes accompanying GB recurrence. For the first time, the present study examined the potential link of nuclear GARP expression with patient outcomes.

## 2. Materials and Methods

### 2.1. Cell Culture

The human glioblastoma cell line T98G was purchased from the ATCC (CRL-1690) and was cultured in Minimum Essential Medium Eagle supplemented with 10% FCS, 1% Glutamine, and 0.1% Primocin. The human melanoma cell lines, Mewo and Ma-Mel-19, were obtained from Dr. Daniela Kramer (Mewo, RRID:CVCL_0445, Cellosaurus) in Mainz, Germany, in 2021 and from Dr. Annette Paschen (Ma-Mel-19, RRID:CVCL_A156, Cellosaurus) in Essen, Germany, in 2014. Mewo cells were cultured in Dulbecco’s Modified Eagle Medium supplemented with 10% FCS and 0.1% Primocin. Ma-Mel-19 cells were grown in RPMI 1640 supplemented with 10% FCS, 1% Glutamine, and 0.1% Primocin. T98G, Mewo, and Ma-Mel-19 cells were passed every 2 to 3 days by using Trypsin-EDTA. The cell lines T98G and Ma-Mel-19 were authenticated in August 2022 by PCR single locus technology. The results were compared to the online databases of the DSMZ and Cellosaurus (Eurofins Genomics Europe). Patient-derived GSC lines used in this study were established as previously described and have been well characterized in previous studies, in terms of their stem cell frequency (SCF) and expression of various GSC markers [28,31,32,33]. Additional information regarding their origin, SCF, predominant phenotype (Nestin^+/−^, GFAP^+/−^), and percentage of CD133-positive cells, as well as an exemplary analysis of the GSC markers, CD133, platelet-derived growth factor receptor alpha (PDGFR-α), and aldehyde dehydrogenase 1 family member A3 (ALDH1A3), can be found in Figure S1 [28,33,34]. In brief, excess glioblastoma tumor tissue was obtained from patients operated on at the Department of Neurosurgery of the Johannes Gutenberg University Medical Center Mainz (JG-UMC), with informed consent. The use of tumor tissue for research purposes was approved by the JG-UMC Institutional Review Board (permission 08.06.2017 #837.211.12(8312-F). For GSC isolation, a combined enzymatic and mechanical titration procedure was used as previously described [28]. To promote self-renewal, glioma cells were cultured in serum-free NeuroBasal (NB) medium supplemented with the following factors: B27 supplement (Invitrogen, Darmstadt, Germany) and the recombinant human cytokines, basic fibroblast growth factor 2 (bFGF) (10 ng/mL) and epidermal growth factor (EGF), (20 ng/mL) (Biochrom GmbH, Merck KGaA, Darmstadt, Germany). For in vitro differentiation, cells were subjected to EGF and bFGF withdrawal and assessed for the expression of neural lineage specific markers after 7 days. Self-renewal promoting conditions are hence referred to as “NB+bFGF/+EGF” whereas differentiation is indicated by “NB-bFGF/-EGF” in the manuscript.

### 2.2. Western Blot

Protein preparation and Western blotting were performed as previously described in Müller et al., 2023 [35]. Membranes were probed with the following antibodies: anti-CD133/1 (clone: W6B3C1), anti-PDFGR-α (D13C6) (Cell Signaling, #5241T, Danvers, MA, USA), anti-ALDH1A3 (Thermo Fischer Scientific, MA5-25528, Waltham, MA, USA), anti-p53 (DO-1) (Cell Signaling, #18032), anti-actin (C4) (Santa Cruz Biotechnology, sc-47778, Dallas, TX, USA), anti-glial fibrillary acidic protein (GFAP) (DAKO, Z0334, Santa Clara, CA, USA), anti-p21 (Cell Signaling, #2947), anti-phosphorylated-histone H3 (Ser28) (Cell Signaling, #9713S), anti-HSP70 (Enzo Life Sciences Inc., Farmingdale, NY, USA), anti-mouse IgGκ light chain-binding protein horseradish peroxidase (Santa Cruz Biotechnology, sc-516102), goat anti-rabbit IgG H&L horseradish peroxidase (Abcam, ab205718, Cambridge, UK), goat anti-mouse IgG horseradish peroxidase (Santa Cruz Biotechnology, sc-2055), and goat anti-rabbit horseradish peroxidase (Santa Cruz Biotechnology, sc-2054). Signal intensity was analyzed via densitometry (https://imagej.nih.gov, accessed on 17 October 2023) [36].

### 2.3. Flow Cytometry

For flow cytometric analysis, the following fixable viability dye and antibodies were used: FVD506 (eBioscience #65-0866-14, San Diego, CA, USA), anti-GARP (Miltenyi #130-103-820 and 130-103-890, updated ordering numbers: 130-125-511 and 130-125-532, Bergisch Gladbach, Germany), anti-CD133 (epitope AC133, Miltenyi # 130-113-111), and their respective isotype controls (Miltenyi #130-113-434 and Miltenyi #130-113-200). Cells were stained with fixable viability dye prior to surface antibody staining of anti-GARP and anti-CD133. Cells were not fixed for the analysis.

Extensive validation of the anti-GARP antibodies mentioned above and a demonstration of their specificity can be found in Figures S2 and S3 as well as in previous work by Zimmer et al., 2019 [7]. In more detail, the anti-GARP antibodies from Miltenyi were validated against two other flow cytometry certified antibodies (Biolegend, 352506, San Diego, CA, USA; Origene, TA337028, Rockland, MD, USA) (Figure S3) and against the polyclonal anti-GARP antibody used in this study (Origene, AP17415PU-N) (Figure S2). Antibody specificity was demonstrated using GARP-overexpressing Mewo cells, resulting from transient transfection using the LOX-IMVI Cell Avalanche Transfection Reagent (EZ Biosystems, EZT-LOXI-1, College Park, MD, USA) as well as a LRRC32 overexpression plasmid (Origene, SC116699) and an empty vector control plasmid (Origene, PS100001) (Figure S3). Transfection was performed in accordance with the manufacturer’s recommendations. Cells were stained with fixable viability dye and for surface GARP as described above 48 h post-transfection.

Flow cytometry was performed on a BD LSRII flow cytometer (Heidelberg, Germany) and was analyzed using Cytobank [37]. Doublets, debris, and dead cells were excluded from analysis (Figure S4).

### 2.4. Confocal Microscopy

Confocal imaging was performed on a Leica SP8 with HyD detector (Wetzlar, Germany) at the Imaging Core Facility (ICF) of the Forschungszentrum für Immuntherapie (FZI) of the University Medical Center Mainz as described before [7]. The following antibodies were used in the study: anti-nestin (Abcam, ab22035), anti-GARP (Origene, AP17415PU-N), and secondary antibodies goat anti-mouse Alexa Fluor 488 or goat anti-rabbit Alexa Fluor 555 (both Thermo Fisher Scientific, Waltham, MA, USA). Validation and specificity of the anti-GARP antibody (Origene AP17415PU-N) for its use in confocal microscopy can be found in Figure S5 and in previous work by Zimmer et al., 2019 [7].

### 2.5. Animal Experiments

Animal experiments were performed at the Translational Animal Research Facility (TARC) of the JG-UMC, Germany, in accordance with the guidelines of the European Convention for the Protection of Vertebrates Used for Scientific Purposes and under the approval of the State Office of Chemical Investigations of Rhineland-Palatinate (permission #23 177-07/G12-1-020). Immunodeficient mice (strain NMRI) were purchased from a commercial supplier (Charles River Laboratories Germany). After an adaptation period of one to two weeks, mice were subjected to intracerebral injection of GSCs using a standardized procedure as described previously [34,38]. In brief, single-cell suspensions were prepared from glioma sphere cultures by using a combined trypsin/mechanical titration procedure. Cells were washed twice in PBS and re-suspended in PBS at 2 × 10^4^ cells/μL. Cell viability was determined by trypan blue staining. Single-cell suspensions were injected at 5 μL into the caudato-putamen of the right hemisphere using a stereotactic frame (TSE Systems, Bad Homburg, Germany) and the following stereotactic coordinates in reference to the bregma: 1 mm (anteroposterior axis), 3 mm (lateromedial axis), 2.5 mm (vertical axis). Mice were sacrificed at the first manifestation of tumor-associated neurological symptoms.

### 2.6. GARP Immunohistochemistry and Immunofluorescence

Tumor-bearing mouse brains were extracted and fixed in 4% paraformaldehyde in PBS for at least 24 h at 4 °C as described previously [38]. Briefly, after fixation, brains were paraffin-embedded, dissected into 1–3 μm thick coronal sections and analyzed by immunohistochemical or immunofluorescence staining using antibodies specific to human nestin (R&D Systems GmbH, Wiesbaden-Nordenstadt, Germany), GFAP (DAKO, Z0334), or GARP (Origene, AP17415PU-N). Previous work has demonstrated the specificity of the anti-GARP antibody (Origene, AP17415PU-N) for its use in immunohistochemistry and immunofluorescence [7,39]. For analysis, ImageJ2 (Available online: https://imagej.net/ImageJ2, accessed on 16 August 2021) was used [40].

A GB patient cohort from Zimmer et al., 2019 [7], was reanalyzed to correlate the frequency of GARP^NU+^ cells in tumor tissue to patient overall survival regardless of IDH status. Patient characteristics are described in detail in Figure 1 of Zimmer et al., 2019 [7]. In brief, the patient cohort consisted of 35 newly diagnosed (WHO stage IV) GB patients from the Department of Neurosurgery in Idar-Oberstein, Germany, between January 2009 and May 2015. The median high and low survival times were 12 and 4 months. Primary tumor tissue was resected and stained for GARP via immunohistochemistry. Description of the immunohistochemical staining process can be found in Zimmer et al., 2019 [7]. The frequency of GARP^NU+^ was semi-quantified in tumor tissue with regions of labeled nuclei (categorized as >90%, >50%, >10% GARP^NU+^ cells) at the Institute of Neuropathology, University Medical Center Mainz, Germany [7].

### 2.7. Cell Sorting

Single-cell suspensions of the GSC line, #1095, were stained sequentially with the following: fixable viability dye FVD780 (eBioscience #65-0865-14), unconjugated anti-GARP antibody (Origene, AP17415PU-N) or a control unconjugated IgG rabbit isotype antibody (R&D Systems, AB-105-C), followed by a PE-conjugated goat anti-rabbit secondary antibody (Invitrogen, P2771MP). Cells were sorted into GARP^low^ and GARP^high^ populations. Cell sorting gates were defined as the lower 10th (GARP^low^) and upper 90th percentiles (GARP^high^) of all cells. An example gating strategy and proof of positive GARP staining can be found in Figure S6. Debris, doublets, and dead cells were excluded from analysis. Sorting was performed using BD Aria II and III cell sorters at the Core Facility Flow Cytometry (CFFC) of the Forschungszentrum für Immuntherapie (FZI) of the University Medical Center Mainz.

### 2.8. Extreme Limiting Dilution Assay

The self-renewal capacity of GSC lines was analyzed by extreme limiting dilution assay (ELDA). In brief, single-cell suspensions were serially diluted in self-renewal promoting medium (NB+bFGF/+EGF) and seeded into 24 well plates. The number of replicates used for each serial dilution are indicated as follows: 12 for 100 cells/well, 18 for 50 cells/well, 24 for 25 cells/well, 58 for 12.5 cells/well, 24 for 6.25 cells/well, 18 for 3.125 cells/well, and 12 for 1.56 cells/well. Cells were incubated for three weeks to develop neurospheres. Wells were assessed for neurosphere formation; a positive result was recorded for each dose (number of seeded cells/well) if the examined well contained at least one neurosphere. Each experiment was repeated independently three times. Stem cell frequency (SCF) was calculated using the ELDA: Extreme Limiting Dilution Analysis webtool from the Walter and Eliza Hall Institute of Medical Research (https://bioinf.wehi.edu.au/software/elda/, accessed on 6 September 2023) [41].

### 2.9. Bioinformatic Pipeline

In a previous work, Kim et al., 2020, performed Illumina RNA-Sequencing on a total of 155 glioblastoma samples derived from 28 patients [32]. These consisted of primary, recurrent, and secondary recurrent tumors (128 samples) as well as GSC cultures developed from freshly resected tumor tissue (27 samples). We obtained unnormalized gene counts through the Gene Expression Omnibus database (GEO) under the accession number: GSE139533. Gene counts were normalized with DESeq2 and analyzed using the likelihood ratio test to decipher the effect of progressing tumor stages on transcript levels within the same patient [42]. Normalized counts for CD133 and GARP were plotted with GraphPad Prism version 9.3.1 for Windows, GraphPad Software, San Diego, CA, USA, www.graphpad.com.

Survival analysis of CD133 and GARP was performed using OncoLnc (http://www.oncolnc.org/, accessed on 8 July 2023) which is based upon data generated by The Cancer Genome Atlas (TCGA) Research Network (https://www.cancer.gov/tcga, accessed on 8 July 2023) [43,44,45].

### 2.10. Statistics

Statistical analysis was performed with Student’s *t*-test, the likelihood ratio test, the chi-squared test, or two-way ANOVA as indicated. Data are displayed as mean values ± SEM or ±SD as indicated. Survival curve comparison was analyzed using the log-rank (Mantel–Cox) test using GraphPad Prism. Statistical significance is indicated as follows: * *p* < 0.05, ** *p* < 0.01, *** *p* < 0.001, **** *p* < 0.001, and ns (not significant).

## 3. Results

### 3.1. GARP Expression Is Conserved across Different Types of GSCs In Vitro and In Vivo

**Figure 1:**
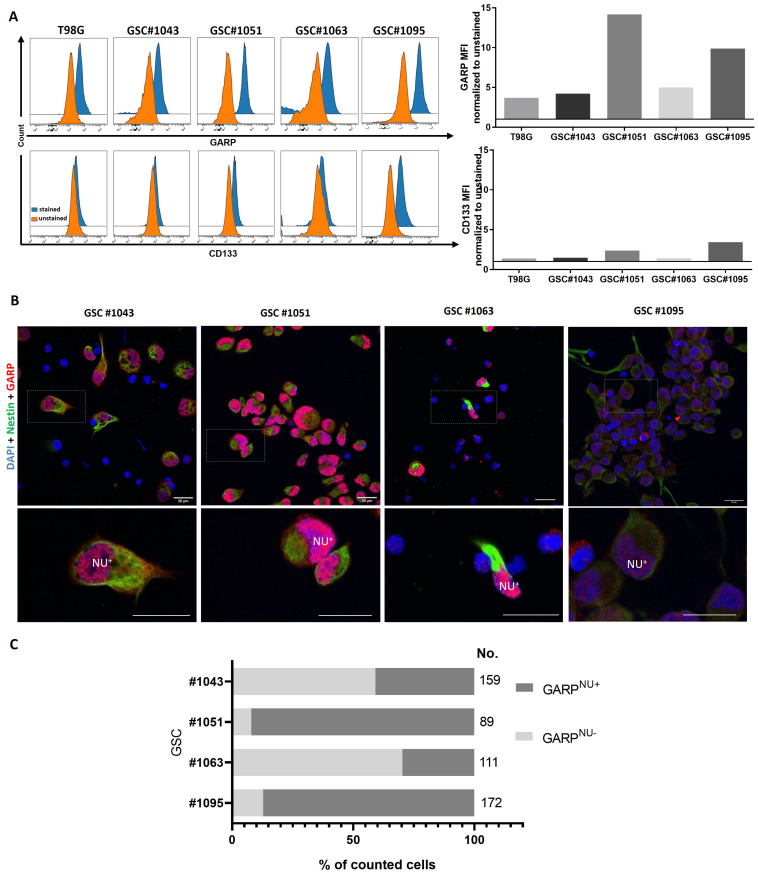
(**A**) Flow cytometric analysis of surface GARP and CD133 on different GSCs and the control, non-stem, GB cell line, T98G. Doublets, debris, and dead cells were excluded from the analysis. Mean fluorescence intensity (MFI) was normalized to the MFI of the respective unstained control. (**B**) Confocal images of GARP- and nestin-expressing GSCs and T98G. Cells were stained for GARP (red) and nestin (green). Cells were counterstained for their nuclei with Hoechst (blue). Note the intranuclear localization of GARP (NU^+^). Scale bar corresponds to 20 µm. (**C**) Percentage of GARP^NU+^ cells were determined by counting GARP stained nuclei. “No.” indicates the number of counted cells for the analysis.

We have previously shown that GARP is expressed by three human GSC lines and by the conventional human glioblastoma cell line, T98G [7]. The questions that remained were whether GARP expression is restricted to a particular type of GSCs or if it represents a common phenotypic trait shared by different subsets of GSCs. To address these questions, we analyzed the expression of GARP in a panel of heterologous GSC lines, differing in their self-renewal capacity, degree of differentiation, and expression of CD133, a proposed marker for GSCs in the past (Figure S1A). All GSCs used in this study invariably expressed nestin, a neural stem cell marker, but they varied in their expression of the astrocyte differentiation marker, GFAP, and CD133, a putative GSC marker (Figure S1A). A non-stem glioblastoma cell line, T98G, (ATCC CRL-1690) was analyzed in parallel as a control. Flow cytometry revealed that the surface expression of GARP varied across heterologous GSCs (Figure 1A). Notably, variations in GARP expression paralleled variations in CD133 levels indicating that GARP and CD133 are not mutually exclusive markers (Figure 1A). Line-dependent variations in GARP expression were also confirmed by microscopic evaluation of intracellular GARP (Figure 1B,C). Confirming our previous observations, microscopic analysis revealed that GARP localization in GSCs is not restricted to the cell membrane, a normal localization site for GARP, but it also extends to the nuclear compartment (Figure 1B) [7,30]. The nuclear localization of GARP was evident in confocal microscopy with co-staining for nestin, an established marker of neural stem/progenitor cells expressed in the cytoplasm. The prevalence of cells with nuclear GARP (termed hereafter as “GARP^NU+^”) varied between different GSC lines (Figure 1B,C) and mirrored the levels of surface-expressed GARP (Figure 1A), indicating a possible relationship between the two forms. For example, the GSC lines #1051 and #1095 had the highest levels of surface-membrane-associated GARP (Figure 1A), and they also exhibited a high proportion of cells with GARP^NU+^ (92.1% and 87.2%, Figure 1B,C). Vice versa, GSCs with moderate levels of surface GARP (#1043 and #1063, Figure 1A) had lower proportions of cells with GARP^NU+^ (40.9% and 29.7%, Figure 1C).

Our in vitro findings prompted us to test if GARP/GARP^NU+^ expression is sustained in vivo in GSCs involved in tumor propagation. To this end, we analyzed xenograft tumors grown from two GSC lines that express the lowest (line #1043) and highest (line #1051) levels of GARP in vitro (Figure 1). Both lines gave rise to highly invasive brain tumors as ascertained by immunohistochemical staining with an antibody specific for human nestin (Figure S7) and had comparable rates of tumor growth [34]. Immunofluorescence staining for GARP revealed its expression in both #1051 and #1043 xenografts (Figure S8). Notably, GARP expression in #1043 xenograft (low expressor in vitro, Figure 1) was comparable with that in #1051 xenograft (high expressor in vitro, Figure 1), suggesting that GARP expression in GSCs might be even more profound in the tumor context. Concordant with our in vitro findings, tumor-propagating GSCs also showed GARP localization in both the cytoplasm and nucleus (Figure S8). Additionally, GARP^NU+^ was observed to be co-expressed with nestin (Figure S8). These results further support the conclusion that GARP/GARP^NU+^ expression might be a common trait stably sustained (or even augmented) in GSCs involved in tumor propagation.

### 3.2. Intratumoral Heterogeneity of Subcellular Distribution Patterns of GARP

GBs are known for their high degree of intratumoral heterogeneity, which is thought to reflect the hierarchical diversity of cellular states generated by GSCs [12,46]. Our observation that heterologous GSCs vary in their levels of GARP/GARP^NU+^ (Figure 1 and Figure S8) prompted us to check if this is a mere reflection of intertumoral diversity, GARP/GARP^NU+^ association with a particular GSC subtype or cellular state, or a hierarchical diversification taking place during tumor growth. To address these questions, we made use of isogenic GSCs (lines IT-726-#1, IT-726-#2, IT-726-#3a, IT-726-#3b, and IT-726-#4) that have been isolated from different regions of the same tumor (Figure S9—Cohort 2”—comparison line 1) and provide a unique model for analyzing the impact of intratumoral heterogeneity in an isogenic background [31,32]. Indeed, despite their identical genetic background, isogenic GSCs from the IT-726 set displayed notable morphological differences, considerable variations in their self-renewal capacity, and expression of GSC-associated markers CD133, ALDH1 A3, and PDGFR-α (Figure S1) [31,32].

**Figure 2:**
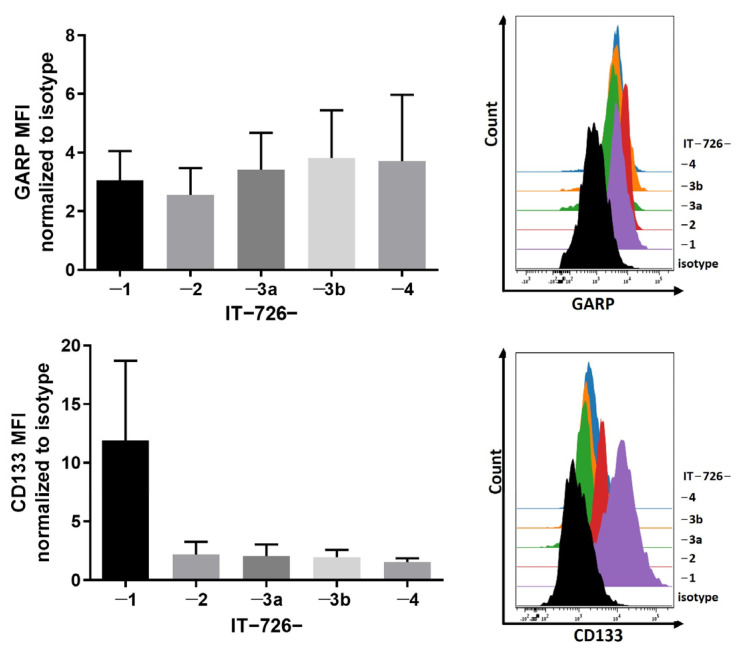
GARP expression in isogenic GSCs derived from newly diagnosed GB IT-726. Flow cytometric analysis of IT-726-1, -2, -3a, -3b, and -4. Doublets, debris, and dead cells were excluded from analysis. Mean fluorescence intensity (MFI) was normalized to the MFI of the unstained control. Histograms display one representative result of three independent measurements. Data are displayed as mean values ± SEM.

**Figure 3:**
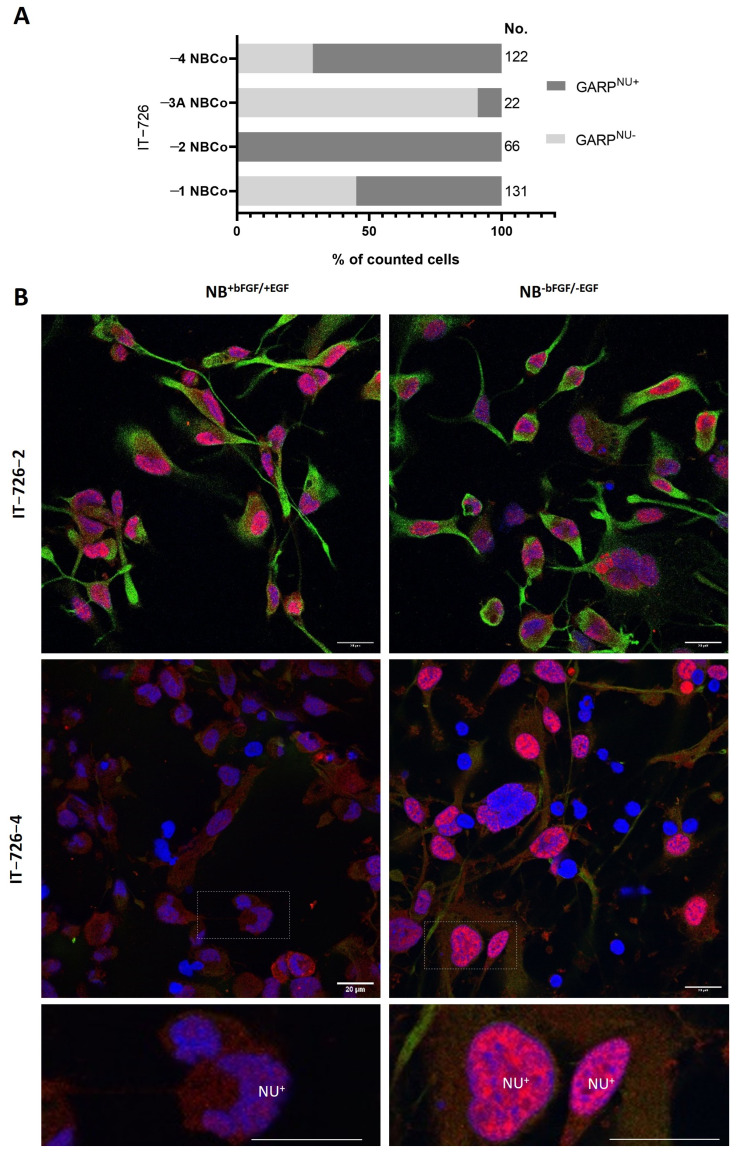
Analysis of expression and localization of GARP in isogenic GSC cell lines, which vary in differentiation states derived from different regions of the same tumor. (**A**) Number of GARP positive nuclei for GSC lines IT-726—1, -2, -3A, and -4 were analyzed by counting double positive (Hoechst and GARP) cell nuclei (NU^+^). “No.” indicates the number of counted cells for the analysis. (**B**) Confocal images of GARP- and nestin-expressing GSC IT-726 -2 and -4. Cells were stained for their nuclei with Hoechst (blue), GARP (red), and nestin (green). Note the intranuclear localization of GARP. Scale bar corresponds to 20 µm.

Interestingly, we found no apparent correlation between CD133 expression and the degree of self-renewal activity. For example, the lines IT-726-1 and IT-726-3B had comparable degrees of self-renewal activity (Figure S1A), but they differed profoundly in the expression of surface CD133 (glycosylated epitope AC133) (Figure 2). Vice versa, the line IT-726-4 expressed similar levels of surface CD133 as the lines IT-726-2, IT-726-3A, and IT-726-3B, (Figure 2), but it stood out markedly from the other lines in terms of its extremely low self-renewal capacity (Figure S1A). In contrast to CD133, the expression of surface GARP was very similar across isogenic lines, and it did not parallel the striking difference in CD133 expression between the IT-726-1 line and its isogenic counterparts (Figure 2). In comparison to the uniform expression of surface GARP, the patterns of GARP subcellular distribution between IT-726 lines were heterogeneous, with the proportion of GARP^NU+^ cells varying across different isogenic lines (Figure 3A). The highest level of GARP^NU+^ was found in line IT-726-2, which had a prominent expression of nuclear GARP in nearly every cell (Figure 3B, IT-726-2 upper panel). GARP expression was also examined on IT-726 cell lines grown in self-renewal-promoting (NB+bFGF/+EGF) versus differentiation-promoting (NB-bFGF/-EGF) conditions. Interestingly, IT-726-2 displayed a prominent expression of nuclear GARP in almost every cell regardless of culture condition. In contrast, other isogenic IT-726 lines exhibited a mixed pattern of GARP localization in both nuclear and cytoplasmic compartments in self-renewal-promoting conditions (NB+bFGF/+EGF) (Figure 3B, shown for IT-726-4). Notably, the nuclear localization of GARP appeared to be more profound when cells were grown in differentiation-promoting conditions (NB-bFGF/-EGF), suggesting an inverse correlation between GARP^NU+^ and self-renewal capacity. The IT-726-2 line, in which the GARP^NU+^ pattern was predominant (Figure 3B), had a lower self-renewal capacity when compared to the other isogenic counterparts (Figure S1A), consistent with this interpretation.

### 3.3. Relationship between GARP Expression and GSCs Stemness

**Figure 4:**
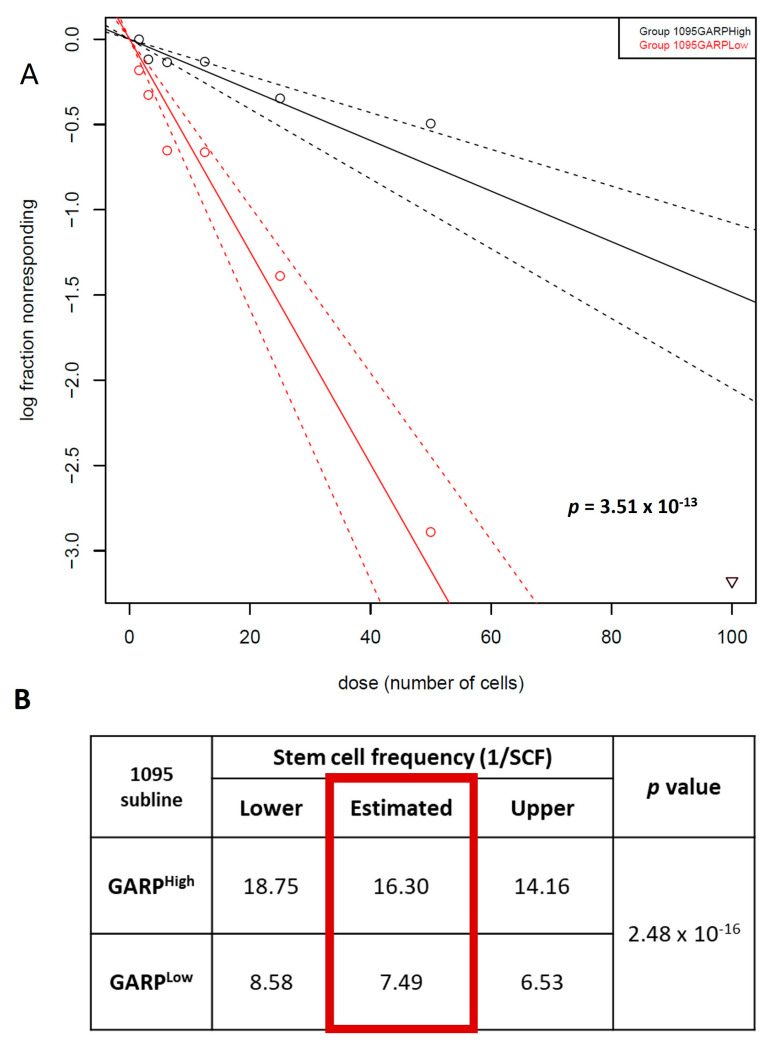
Quantitative assessments of self-renewal capacity by extreme limiting dilution assay (ELDA). (**A**) Representative results. (**B**) The pooled results from three independent experiments are indicated in the table. GARP^high^ and GARP^low^ correspond to isogenic GSCs differing in their GARP expression, which were FACs sorted from the GSC line #1095. Estimates of the stem cell frequency (SCF) are framed in red, while lower and upper indicate the confidence intervals for 1/SCF. Statistical significance between groups was calculated by chi-squared tests.

The dual capacity of self-renewal and differentiation are the fundamental and unique properties of stem cells. We therefore sought to determine if there is an association between GARP expression and self-renewal. To this end, cell populations differing in GARP expression (GARP^high^ or GARP^low^) were FACS sorted from the GSC line #1095 and compared with respect to self-renewal activity by ELDA. A demonstration of the sorting efficacy and quantification of surface GARP expression on GARP^high^ vs. GARP^low^ sorted cells via flow cytometry can be found in Figure S6A. The GSC line #1095 was chosen for these investigations because of its well-established stemness attributes as well as molecular and cellular responses to clinically relevant treatments in vitro and in vivo [28,33,34,35]. The results of ELDA assessments revealed that GARP^high^ and GARP^low^ populations of GSCs differ in their self-renewal propensity, which was significantly (*p* = 2.48 × 10^−16^) lower in the GARP^high^ subpopulation compared to GARP^low^ subpopulation (Figure 4).

**Figure 5:**
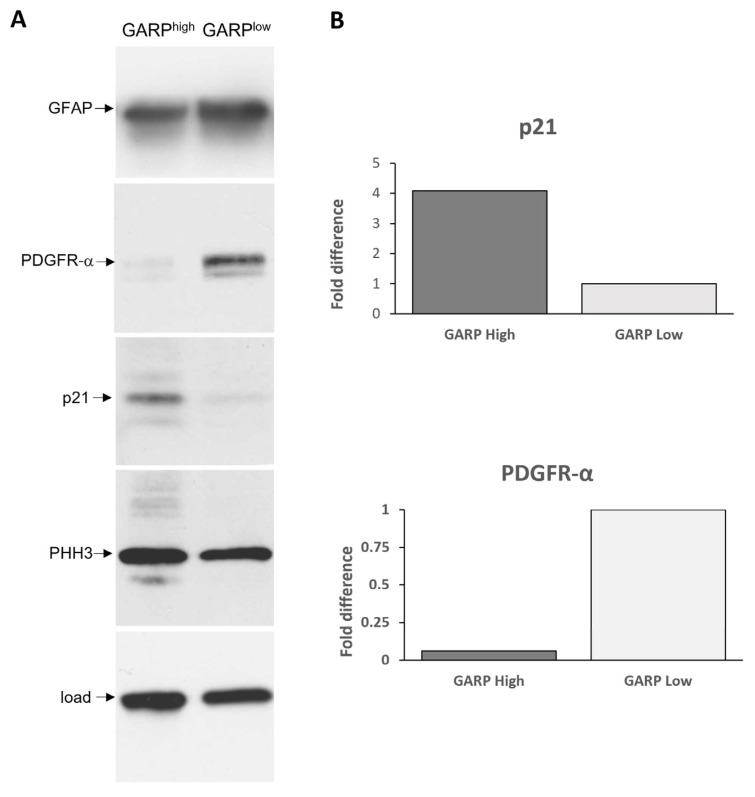
Comparative assessments of glial fibrillary acidic protein (GFAP), platelet-derived growth factor receptor alpha (PDGFR-α), p21, and phosphorylated histone H3 (PHH3) in FACs-sorted GARP^high^ and GARP^low^ isogenic GSCs (#1095) by Western blot. (**A**) Representative results. HSP70 was used as a loading control. The following antibodies were used to probe the membranes: anti-GFAP (DAKO, Z0334), anti-PDFGR-α (D13C6) (Cell Signaling, #5241T), anti-p21 (Cell Signaling, #2947), anti-phospho-histone H3 (Ser28) (Cell Signaling, #9713S), anti-HSP70 (Enzo Life Sciences Inc.), anti-mouse IgGκ light chain-binding protein horseradish peroxidase (Santa Cruz Biotechnology, sc-516102), and goat anti-rabbit IgG H&L horseradish peroxidase (Abcam, ab205718). (**B**) PDGFR-α and p21 bands were quantified by densitometry.

As loss of self-renewal is a prerequisite for stem cell differentiation, the outcome of the ELDA experiments raised the possibility that GARP expression may be related to differentiation of GSCs. To address this question, GARP^high^ and GARP^low^ GSCs were subjected to comparative assessments for the differentiation-inducing factor p21 and the differentiation-associated markers, GFAP and PDGFRα, activated during astrocyte or oligodendrocyte differentiation. The results showed that GARP^high^ GSCs had considerably higher steady-state levels of p21 compared to GARP^low^ GSCs, which seems consistent with the interpretation that increased expression of GARP is associated with a more differentiated state. However, an elevated level of p21 was unaccompanied by increased expression of GFAP or PDFGRα in GARP^high^ GSCs. Quite the contrary, the expression of either GFAP or PDFGRα was found to be lower in GARP^high^ GSCs than in GARP^low^ GSCs (Figure 5) with the difference in PDGFRα levels being especially profound (Figure 5B). Although the difference in GFAP expression between GARP^high^ and GARP^low^ GSCs was less profound, it was also confirmed by using a different approach, namely the estimation of GFAP-positive differentiating cells by immunofluorescence staining (Figure S10). A decline in proliferative activity is an important functional hallmark of normal stem cell differentiation. Deviating from this rule, GARP^high^ GSCs, which had a reduced self-renewal capacity in comparison to GARP^low^ GSCs (Figure 4), had comparable levels of the proliferation marker PHH3 (Figure 5). Collectively, our data indicate that increased expression of GARP correlates with reduced self-renewal but not with the cessation of proliferation or induction of phenotypic traits of neural differentiation.

### 3.4. GARP mRNA and Surface Protein Levels Do Not Predict GB Patient Survival

**Figure 6:**
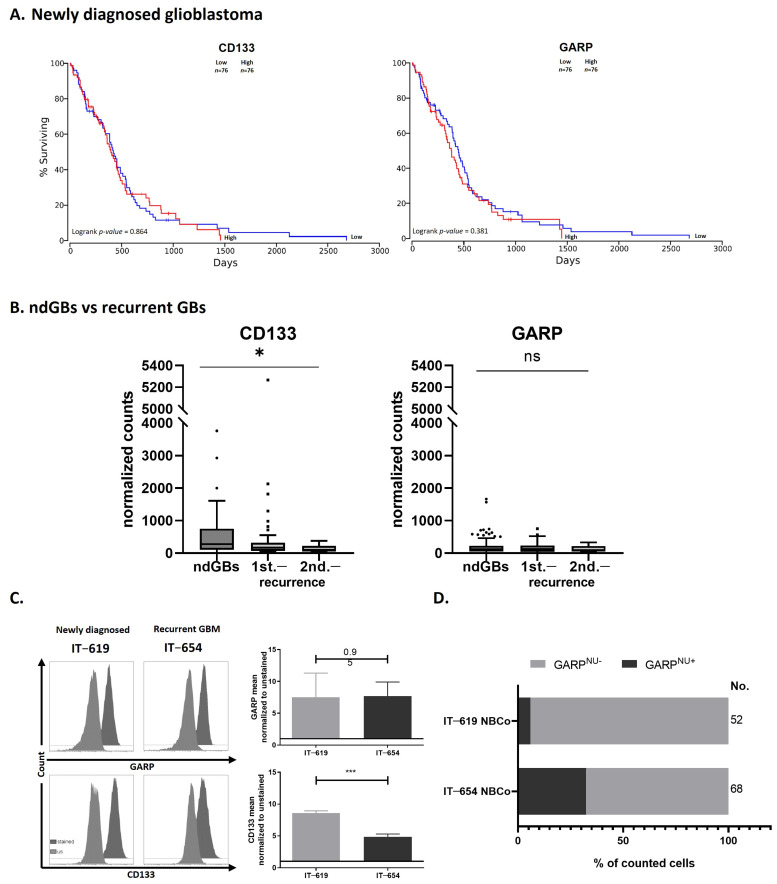
GARP expression in GB is unaffected throughout therapy. (**A**) Survival analysis of GARP and CD133 based on data available through The Cancer Genome Atlas (TCGA). GARP and CD133 mRNA expression data of 152 primary glioblastomas were divided 50/50 into either “low” expression or “high” expression and were analyzed for patient survival. (**B**) Retrospective analysis of transcriptomic data of 155 GB samples from 28 patients of Kim et al., 2020 [32]. ndGBs, first, and second recurrent tumors were analyzed for their GARP and CD133 mRNA levels across tumor stages. (**C**) Flow cytometric analysis of IT-619 and IT-654. Doublets, debris, and dead cells were excluded from analysis. Recurrent IT-654 GSCs exhibited stable surface GARP levels after TMZ and radiotherapy, whereas expression of CD133 decreased after treatment. The MFIs were normalized to the unstained control. *n* = 3. Significance was calculated by Student´s t-test and is indicated as follows: * *p* < 0.05, *** *p* < 0.001, and ns (not significant). (**D**) Number of GARP-positive nuclei for GSC IT-619 and IT-654 were analyzed by counting double-positive (Hoechst and GARP) cell nuclei. “No.” indicates the number of counted cells for the analysis.

Having established that GARP is expressed in patient-derived GSCs, we sought to determine whether a correlation exists between GARP expression and GB patient survival. To address this question, gene expression and survival data from the TCGA database were analyzed for GARP and CD133 by using OncoLnc.org (Figure S9—Cohort 1) [43,44,45]. Based on the TCGA dataset, consisting of 152 patients with newly diagnosed glioblastomas, all patients analyzed were stratified by their expression levels of GARP into either “GARP-high” (upper 50%) or “GARP-low” (lower 50%) groups and were analyzed for their survival rates via the online tool OncoLnc [43]. We could not find any significant difference in survival between GARP-high and GARP-low groups (Figure 6A). Similarly, no significant correlation was found between survival and CD133 expression (Figure 6A). In a second approach, GARP and CD133 transcript levels were compared between newly diagnosed glioblastomas (ndGB) and progressed recurrent glioblastomas (recGB) as depicted in Figure S9—Cohort 2—comparison line 2. To this end, we retroactively analyzed RNAseq data from a database that compiles RNAseq data for ndGBs (23 patients) or recGBs (21 patients) as well as 27 primary cultures derived from either ndGB (ndGB-GSCs, 17 cultures) or recGB (recGB-GSCs, 10 cultures) [34]. GARP and CD133 mRNA expression were compared between ndGB samples (ndGB tissues and ndGB-GSC cultures) and recGB samples (recGB tissues and recGB-GSCs). The results showed that expression levels of GARP do not differ significantly between ndGB and recGB samples whereas CD133 levels were found to be significantly reduced in recGB samples compared to ndGB samples (Figure 6B). In a third approach, surface GARP expression was compared between isogenic ndGB-GSCs and recGB-GSCs isolated from ndGB and recGB tumors of the same patient (Figure S9—Cohort 2—comparison line 3). Both ndGB-GSCs and recGB-GSCs showed virtually the same levels of surface GARP expression, whereas the level of CD133 was significantly lower in recGB-GSCs in comparison to ndGB-GSCs (Figure 6C). This agreed with the results of the RNAseq analysis (Figure 6B) as both GARP transcript and surface GARP (Figure 6C) levels were consistently expressed regardless of disease progression. Interestingly, in contrast to GARP transcript and surface GARP levels, it was found that the percentage of GARP^NU+^ cells were elevated in the recurrent GSC line, IT-654 (Figure 6D).

Collectively, these results obtained via different experimental approaches indicate that expression of GARP mRNA and surface protein remain at a constant level throughout GB progression and after therapy—in contrast to the fluctuating expression of CD133. This sustained expression of surface GARP and GARP transcript levels in ndGBs and recGBs suggests the potential utility of GARP as a reliable GSC biomarker, which persists at different tumor stages, possibly allowing for the detection of potential residual disease of a remarkably invasive cancer type.

### 3.5. Nuclear Localization of GARP Correlates with Poor Survival in Patients with GB

**Figure 7:**
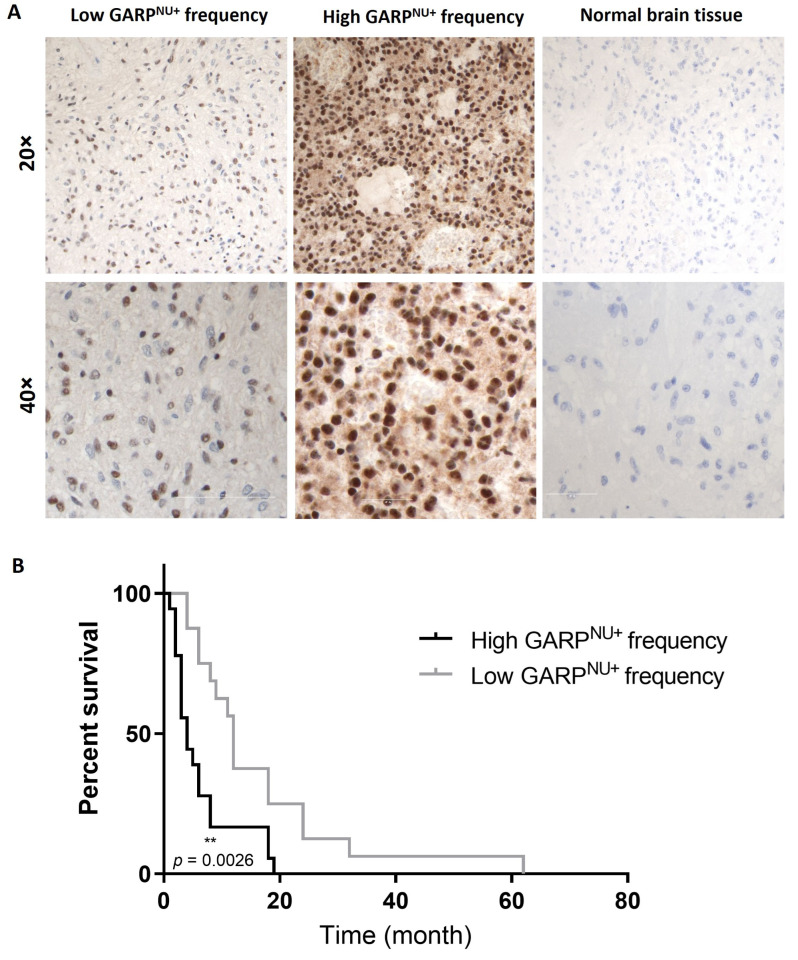
Nuclear GARP is a potential new prognostic biomarker for GB patient survival. (**A**) Immunohistochemistry of GARP in glioblastoma. GB (WHO grade IV) with low frequency of labeled nuclei (magnification ×20 and ×40) and GB with palisading necroses and a high frequency of stained nuclei (magnification ×20 and ×40). Normal brain tissue had no detectable GARP expression. Bar corresponds to 50 µm (40×) and 200 µm (20×), respectively. (**B**) Survival analysis of 35 GB patients based on their GARP-positive nuclei counts (1: high frequency, *n* = 16 and 2: low frequency, *n* = 19). Comparison of survival curves was performed by log-rank (Mantel–Cox) test (** *p* < 0.01).

As we observed an upregulation of the percentage of GARP^NU+^ cells in the recurrent GSC line, IT-654 (Figure 6D), we wanted to explore a possible link between GARP^NU+^ and the survival rate of GB patients (Figure S9—Cohort 3). Therefore, we retroactively assessed GARP^NU+^ levels in tumor tissue from a cohort of 35 newly diagnosed GB patients (WHO stage IV) and correlated them to patient overall survival (Figure 7, representative images) [7].

Notably, all GB patients in the cohort were found to express GARP^NU+^ but varied in their frequency of GARP^NU+^ cells. Therefore, we divided the cohort into two groups based on their frequency of GARP^NU+^ expression. The first group encompassed 19 GB patients with tumors having a low frequency (~50%) of GARP^NU+^ cells. The other group included 16 patients with a high frequency (>90%) of GARP^NU+^ cells. In striking contrast with the transcriptomic analysis, which showed a consistent lack of correlation between GARP mRNA levels and patient survival (Figure 6A), stratification by GARP^NU+^ revealed a significant correlation between GARP^NU+^ and GB patient survival (Figure 7B). The results showed that patients with a low frequency of GARP^NU+^ had a significantly longer overall survival in comparison to patients with a high frequency of GARP^NU+^ (medians low: 12 months, high: 4 months; *p* = 0.0026, Figure 7B). These results indicate that the abundance of the GARP protein in the nuclear compartment—not GARP transcript levels—is associated with survival in patients with GB.

## 4. Discussion

GSCs comprise a heterogenous and highly volatile group of cells, which can switch between different phenotypes and molecular programs in response to environmental changes. The high degree of phenotypic plasticity displayed by GSCs poses a challenge in the development of GSC-based diagnostic and GSC-targeting therapeutic strategies. The continuing search for GSC-associated markers has led to the identification of several molecules expressed in some but not all subtypes of human GSCs or associated with some but not all cellular states [11,12]. One approach to counterbalance the phenotypic diversity of GSCs is to simultaneously target multiple markers associated with different types of GSCs, in order to increase the diagnostic coverage of the GSC content in a highly heterogeneous milieu of GBs [11,28,32,47,48]. An alternative possibility is that some phenotypic traits may be conserved across heterogeneous GSCs. Our data indicate that expression of GARP/ GARP^NU+^ may be one such trait. We provide several lines of evidence that GARP/GARP^NU+^ is expressed in otherwise phenotypically distinct GSCs (Figure 1 and Figure 3) and persists invariably across different cellular states (Figure 3). In the past, several groups have tried to identify universal GSC markers. One challenge is that most of the previously identified putative markers of GSCs, e.g., CD133, are not universally expressed in all types of GSCs, which limits the diagnostic utility of such markers for estimating GSC content in tumors [26]. In this regard, both surface GARP and GARP^NU+^ expression appear to be invariably expressed in phenotypically distinct GSCs including CD133^+^ and CD133^−^ subtypes, under in vitro and in vivo experimental conditions (Figure 1, Figure 3, and Figure S8) and in different stages of GB progression (ndGBs or recGBs) (Figure 6).

Our data indicate that GSC-associated expression of GARP persists in different cellular states. However, the degree of GARP expression varies between different cellular states. Interestingly, we find that expression of GARP is elevated in the state associated with a reduced self-renewal but not proliferative capacity and in conjunction with loss of differentiation-associated traits (Figure 4, Figure 5 and Figure S10). Such a pattern is reminiscent of the transit-amplifying state during neurogenesis whereby slow-cycling neural stem cells must first exit from the state of self-renewal and convert into more differentiated but uncommitted and fast-proliferating transit-amplifying progenitors, prior to entering the lineage-commitment stage and differentiation [49]. The simultaneous reduction in self-renewal and differentiation-associated traits without loss of the proliferation activity seen in GARP^high^ GSCs suggests that GARP may have a role in GSCs’ transition from the slow-cycling self-renewal state to a more differentiated and proliferation-competent state similar to that of transit-amplifying progenitors. It should be noted that even the complete loss of self-renewal does not lead to a loss of the tumor-propagating capacity of GSCs, and recent evidence indicates that GB propagation is driven primarily not by self-renewing GSCs but their non-self-renewing progenies [50].

It is important to note that GARP expression is not limited to GSCs alone. Cancer cells, including glioblastoma (as shown in this work with T98G in Figure 1A), activated regulatory T cells and B cells, and platelets are all known to express GARP on their surfaces [28]. Therefore, GARP alone cannot be used to identify GSCs but rather in combination with a panel of other markers to better distinguish between GSCs and other GARP-positive cells in the tumor microenvironment. In this regard, our finding that elevated GARP expression coincides with a significant increase in p21 expression suggests that dual assessments for GARP and p21 may enable a distinguishment between GSC and non-GSC cells. Considering that p21 plays important roles in the maintenance of neural stem cells and is one of the factors implicated in GB radioresistance, the concomitant elevation of p21 and GARP in GSCs further supports the potential merits of GARP as a predictive and prognostic biomarker for GB [51,52]. A limitation of this exploratory work is that it mainly analyzed the expression of GARP in comparison to one GSC reference marker, CD133. Future studies are needed to analyze in depth the association of GARP expression with an expanded panel of putative GSC markers to further evaluate how universally and stably expressed GARP is on GSCs, and on different cellular components of the tumor microenvironment, especially those that are known to express GARP, like activated regulatory T cells and platelets.

Intriguingly, whereas GARP mRNA levels are comparable between ndGBs and recGBs, the level of GARP^NU+^ protein correlated with poor survival in patients with GB (Figure 7). Notably, the critical cutoff for GARP^NU+^ was >90% (Figure 7), which is significantly higher than the 10% cutoff for CD133 expression implicated as a predictive marker for GB recurrence [53].

Although the link between a high frequency of nuclear GARP and poor outcomes of GB patients provides a novel and intriguing insight into the previously unsuspected role of GARP^NU^ in GB, it is important to also acknowledge the limitations of this exploratory study. One is the small patient cohort size (*n* = 35). The relationship between GARP^NU+^ and clinical outcome from GB must be validated in future studies using larger datasets. A further confirmation in larger follow-up studies is a prerequisite for the conclusion on the diagnostic value of GARP^NU+^ as a prognostic biomarker for GB.

Cancer stem cells are related to reduced survival in glioblastoma patients [11,12]. Therefore, it was surprising to see that a high frequency of GARP^NU+^ tumor cells was linked to reduced overall survival, despite the observed upregulation of GARP^NU+^ in differentiation-promoting conditions (NB-bFGF/-EGF) (Figure 3B). One possible explanation for this is that elevated GARP levels are linked to enhanced immunosuppression in the tumor microenvironment [7,39]. In more detail, previously, we demonstrated both in melanoma [39] and in glioblastoma [7] that tumor cells upregulate the expression of GARP and thus gain tolerogenic potential. This in turn aids in the suppression of effector CD4^+^ and CD8^+^ T cell function, required for anti-tumor immune responses, and correspondingly induces suppressive regulatory T cells, which further contribute to the suppression of effective anti-tumor immune responses. Furthermore, the upregulation of GARP, an inhibitory protein, upon the differentiation process of cancer stem cells is consistent with previous reports by Ullah et al., 2020, who similarly demonstrated that the immune checkpoints PD-L1 and HLA-G are upregulated by cancer stem cells upon differentiation [54]. The principal binding partner of GARP, TGF-β, has been shown to induce the expression of PD-L1, but it remains unclear if GARP expression can as well [55,56]. It is worth noting that the simultaneous targeting of GARP, TGF-β1, and PD-1 has been shown to be an effective combination therapy, capable of restoring T effector cell function and overcoming resistance to PD-1/PD-L1 blockade [57,58]. Future studies are planned to clarify the relationship between GARP, PD-L1, and differentiation to determine if their contribution to immune suppression is responsible for the observed reduction in patient survival.

Interestingly, we found a discrepancy between RNAseq data from primary tumor tissue samples (Figure 6A) and our histological analysis of the frequency of GARP^NU+^-positive cells in GB patient samples (Figure 7). Whereas no relationship between GARP transcript levels and patient survival was detectable in the TCGA data (Figure 6A), the frequency of GARP^NU+^ GB cells seems to be a suitable prognostic marker for patient survival. It should be considered that the tissue samples used for TCGA RNAseq analysis (Figure 6A) presumably consisted of tumor lysates, which contain a multitude of cell types, ranging from tumor cells to immune cells, up to healthy tissue. As information on the cellular origin of the transcripts is missing due to the bulk sequencing, otherwise significant differences between donors can be diluted into insignificant results based on the individual composition and frequencies of cell types included in the analysis. In addition, GARP mRNA can be detected in many tissues, e.g., heart, kidney, liver, and lung, whereas surface expression of the GARP protein itself seems to be limited to only a number of cell types, e.g., activated regulatory T cells [59], platelets [60], various cancers like GB and malignant melanoma [7,39], and mesenchymal stem cells [61], further contributing to a decreasing validity. Therefore, the identification of the cell type analyzed in RNAseq is key to interpreting and understanding future datasets.

More advanced methods like spatial transcriptomics, multiplex immunofluorescence, and spatial multi-omics single-cell imaging are more fitting to further enhance our understanding of GARP transcript and protein levels in glioblastoma cells and their surrounding microenvironment, as well as their distribution within subcellular compartments [62]. The additional information gained by these techniques would enable the identification of different cell types, their localization within the tumor and relation to other cells of the tumor microenvironment, and the determination of whether a surface or intranuclear localization of the GARP protein is present in these cells. Furthermore, the exclusion of certain cell types (e.g., regulatory T cells or platelets) from the analysis would enable a better understanding of GARP and its subcellular localization on patient outcomes.

Our data suggest that nuclear localization of the GARP protein—rather than abundance of the GARP transcript—is a factor associated with GB progression after therapy. Our finding that GARP is localized to the nucleus is novel and intriguing, as GARP has previously been characterized only as a surface and secreted protein, which currently has no annotated nuclear localization signal (NLS). Interestingly, the use of nuclear localization of an otherwise surface-associated protein as a prognostic marker has been described before [63,64,65]. One such example is the protein Src, which plays a key role in cell morphology, motility, proliferation, and survival [66]. Urciuoli et al., 2017, was able to show in human osteosarcoma that nuclear localization of Src correlates with overall survival and therefore has relevance as a prognostic marker for osteosarcoma patients [64]. Likewise, it has also been described that PD-L1, a T cell inhibitory molecule in cancer, shows a nuclear localization as a reaction to therapy. In more detail, PD-L1 is translocated from the cell surface into the nucleus as a reaction to high-dose doxorubicin therapy regimens. The nuclear localization of PD-L1 was described as a prognostic biomarker, as patients with low PD-L1 nuclear expression had significantly fewer circulating cancer cells and exhibited a longer overall survival [63,65]. While the mechanisms of GARP nuclear localization in GSCs still have to be elucidated, the potential clinical implications of this previously unknown phenomenon are clear given the critical role of GARP in the activation of TGF-β, one of the key factors [67] contributing to GB progression particularly via the maintenance of GSCs via the induction of, e.g., Sox2 and LIF expression [68,69]. Considering that targeting TGF-β-activating ligands in GB has been intensively explored as a promising therapeutic strategy [67,70], the clarification of GARP^NU+^ activities in GSCs may provide novel insights into the interaction of GARP and TGF-β, as TGF-β activation is known to trigger the nuclear localization of proteins like Smad and Smad4 [71]. Further pointing to the potential merit of GARP as a diagnostic and therapeutic target is the dual impact of GARP on cancer progression—via the modulation of regulatory T cells and through the direct activities of GARP exerted on cancer (stem) cells themselves.

## 5. Conclusions

The scope of the present study was to evaluate GARP as a biomarker for heterogeneous GSCs and to determine the effects of GARP on GB patient outcomes. Based on our data, we propose that GARP^NU+^ could potentially serve, in combination with existing GSC markers, as a universal and stably expressed marker for different subsets and cellular states of GSCs as well as a possible prognostic marker for patient outcomes in GB. We propose that GARP assessments may provide the means to identify not only self-renewing GSCs but also their progenies that exit from self-renewal but retain proliferative activity. Further validation of this hypothesis in future studies will require analyses of larger patient cohorts using an extended panel of markers associated with GSCs and GB progression. Future investigations should focus on addressing mechanistic questions, such as the functional significance of GARP in regulating GSC-specific functions, by employing knockdown and/or overexpressing lines, as well as further investigating the role of nuclear GARP, its nuclear retention, and functional relevance.

## Data Availability

Data are available in a publicly accessible repository that does not issue DOIs. Publicly available datasets were analyzed in this study. This data can be found here: https://www.ncbi.nlm.nih.gov/geo/query/acc.cgi?acc=GSE139533 and here: http://www.oncolnc.org/ (accessed on 9 April 2021).

## Supplementary Materials

The following supporting information can be downloaded at: https://www.mdpi.com/article/10.3390/cancers15245711/s1, Figure S1: Heterologous GSC lines differing in their self-renewal capacity, Figure S2: Anti-GARP antibody validation for flow cytometry, Figure S3: Specificity demonstration and validation of anti-GARP antibodies, Figure S4: Flow cytometric gating strategy for GSCs, Figure S5: Anti-GARP antibody validation for confocal microscopy, Figure S6: Flow cytometric gating strategy for GARP^high^ and GARP^low^ sorted GSCs, Figure S7: Invasive xenograft tumors arisen from GSC lines, #1051 and #1043, Figure S8: GARP is expressed in xenograft tumors arisen from GSC lines, #1051 and #1043, Figure S9: Study design and models used for the assessment of GARP, Figure S10: Frequency of GFAP^+^ GARP^high^ and GARP^low^ GSCs.
