# Supplementary material for: Nuclear Glycoprotein A Repetitions Predominant (GARP) Is a Common Trait of Glioblastoma Stem-like Cells and Correlates with Poor Survival in Glioblastoma Patients
Source: Cancers (Basel). 2023 Dec 5;15(24):5711. doi: 10.3390/cancers15245711 (PMC10741777; doi:10.3390/cancers15245711)
Supplement: Supplementary file 1 [file cancers-15-05711-s001.zip › cancers-2701282 images.pdf]

Original Images for Blots (Figure 5)

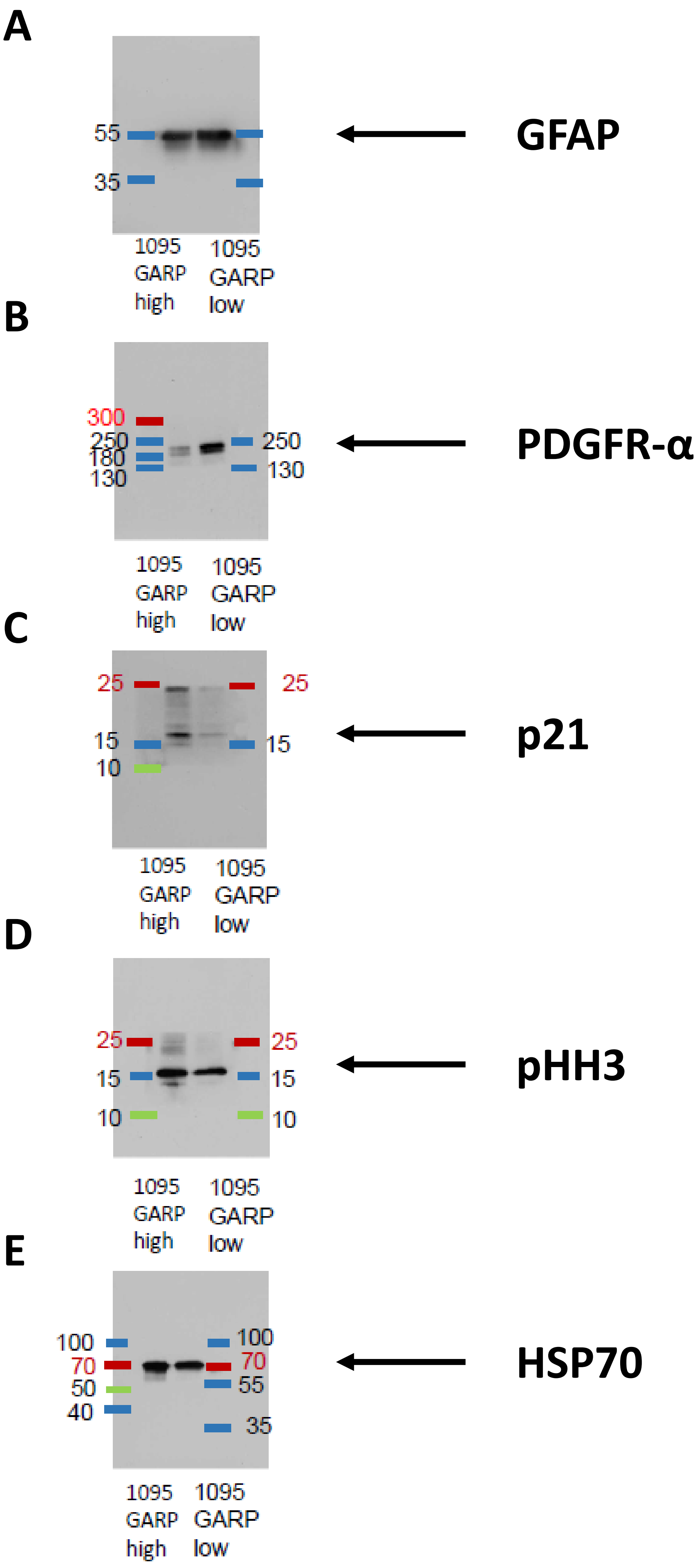

Original Images for Blots (Figure 5): Original, uncropped, and unadjusted blot images shown in Figure 5. The following proteins were analyzed: (A) glial fibrillary acidic protein (GFAP), (B) platelet-derived growth factor receptor alpha (PDGFR-α), (C) p21, (D) phosphorylated histone H3 (PHH3), and (E) HSP70 was used as a loading control. Protein size (kDa) is indicated by the ladders on the left and right sides of the membranes.
