# Supplementary material for: Nuclear Glycoprotein A Repetitions Predominant (GARP) Is a Common Trait of Glioblastoma Stem-like Cells and Correlates with Poor Survival in Glioblastoma Patients
Source: Cancers (Basel). 2023 Dec 5;15(24):5711. doi: 10.3390/cancers15245711 (PMC10741777; doi:10.3390/cancers15245711)
Supplement: Supplementary file 1 [file cancers-15-05711-s001.zip › cancers-2701282-supplementary.pdf]

A

line	origin	SCF	predominant phenotype	CD133, %	ref
#1051	ndGB	1/3	Nestin⁺/GFAP⁻	<1	Barrantes-Freer 2013[29], 2015[26]
#1063	ndGB	1/4	Nestin⁺/GFAP⁻	2.4	Barrantes-Freer 2013[29]
#1095	ndGB	1/4	Nestin⁺/GFAP⁻	<1	Barrantes-Freer 2013[29], 2015[26]
#1043	ndGB	1/3	Nestin⁺/GFAP⁺	<15	this study
IT-726-1	ndGB	1/8	Nestin⁺/GFAP⁺	<80	this study
IT-726-2	ndGB	1/30	Nestin⁺/GFAP⁺	<1	this study
IT-726-3A	ndGB	1/25	Nestin⁺/GFAP⁺	<5	this study
IT-726-3B	ndGB	1/3	Nestin⁺/GFAP⁺	<7	this study
IT-726-4	ndGB	1/59	Nestin⁺/GFAP⁺	<3	this study
IT-619	ndGB	1/15	Nestin⁺/GFAP⁺	>90	this study
IT-654	recGB	1/80	Nestin⁺/GFAP⁺	<70	this study

SCF, stem cell frequency

B

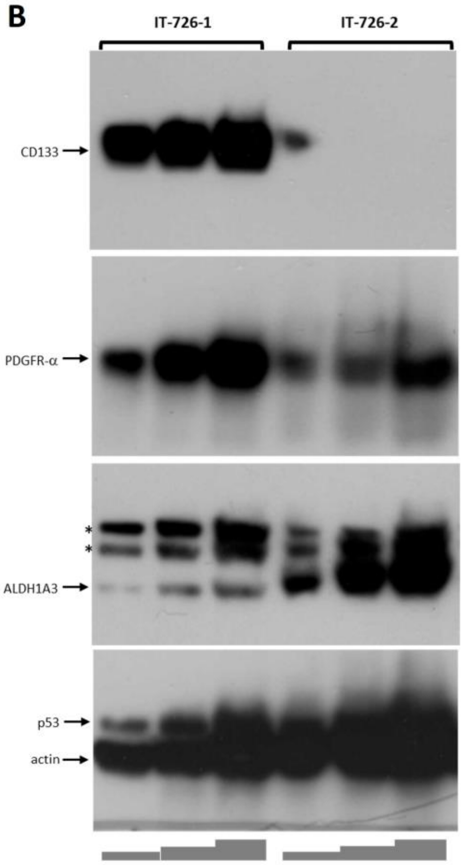

*, unspecific bands

Fig. S1: Heterologous GSC lines differing in their self-renewal capacity. Previous characterization of the heterologous patient derived GSC lines used in this work (A,B) [28,31-33]. Considerable variation in the expression of several GSC markers, including glial fibrillary acidic protein (GFAP), CD133, platelet-derived growth factor receptor alpha (PDGFR-α), and aldehyde dehydrogenase 1 family member A3 (ALDH1A3), was observed in both heterologous and isogenic GSC lines. (B) Example analysis of several GSC markers, including CD133, PDGFR-α, and ALDH1A3, in the isogenic GSC lines, IT-726-1 and IT-726-2 (GSC lines featured in Fig. S1A indicated by red arrows), via western blot using the following antibodies: anti-CD133/1 (clone: W6B3C1), anti-PDFGR-α (D13C6) (Cell Signaling, #5341), anti-ALDH1A3 (Thermo Fischer Scientific, MA5-25528), anti-p53 (DO-1) (Cell Signaling, #18032), anti-actin (C4) (Santa Cruz Biotechnology, sc-47778), goat anti-mouse IgG horseradish peroxidase (Santa Cruz Biotechnology, sc-2055), goat anti-rabbit horseradish peroxidase (Santa Cruz Biotechnology, sc-2054) (B). Cell lysates were loaded in increasing volumes, and actin was used as a loading control.

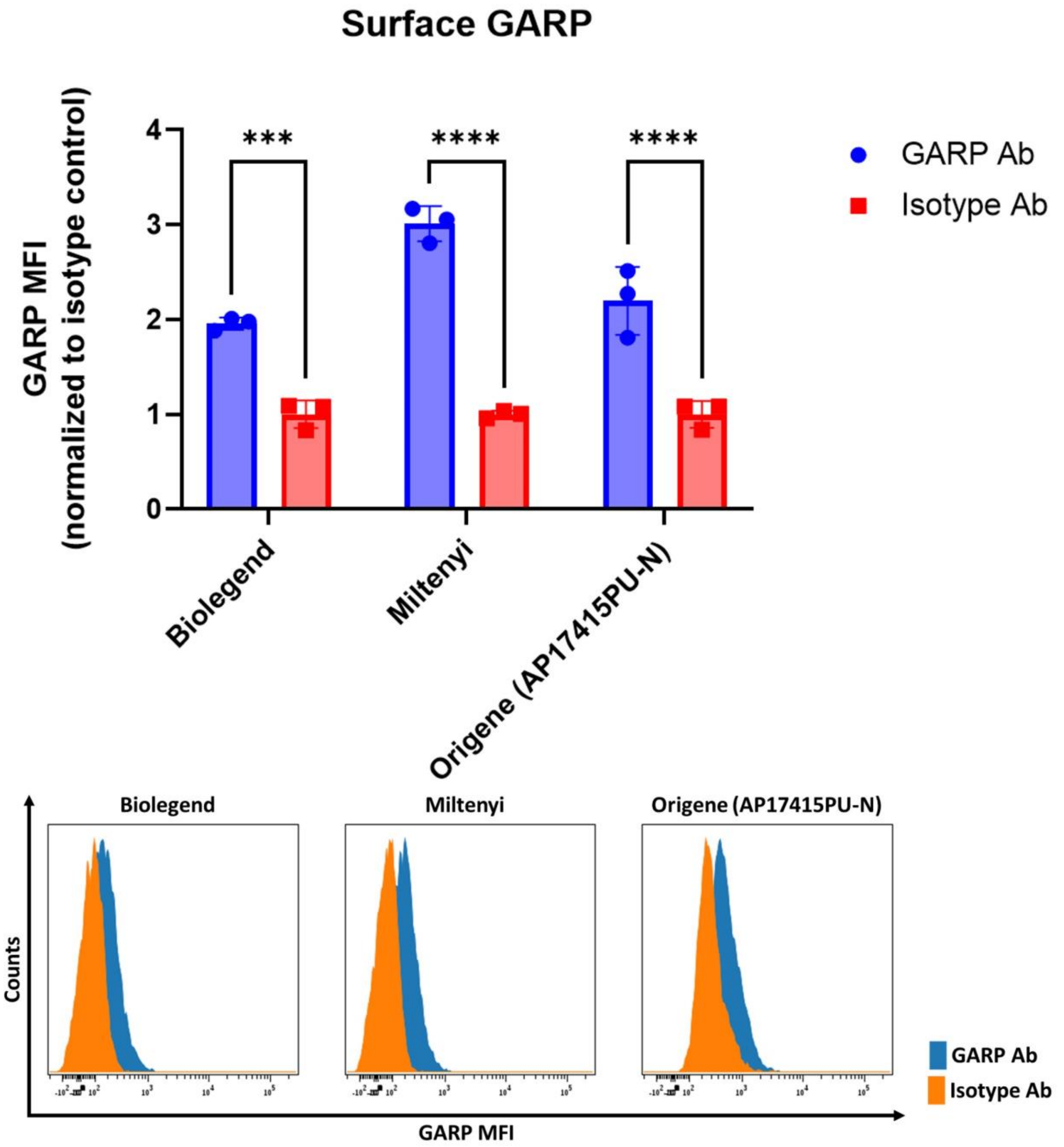

Figure S2: Anti-GARP antibody validation for flow cytometry. Comparative flow cytometric analysis of surface GARP levels on a control human melanoma cell line, Mewo, using three different human anti-GARP antibodies. The following antibodies were analyzed: Miltenyi (130-103-890), Biolegend (352502), Origene (AP17415PU-N). Doublets, debris, and dead cells were excluded from the analysis. Graph shows the mean fluorescence intensity (MFI) normalized to the MFI of the respective isotype control, whereas histograms display one representative result (n=3, ± SD, *** $p < 0.001$, and **** $p < 0.0001$ determined by two-way ANOVA).

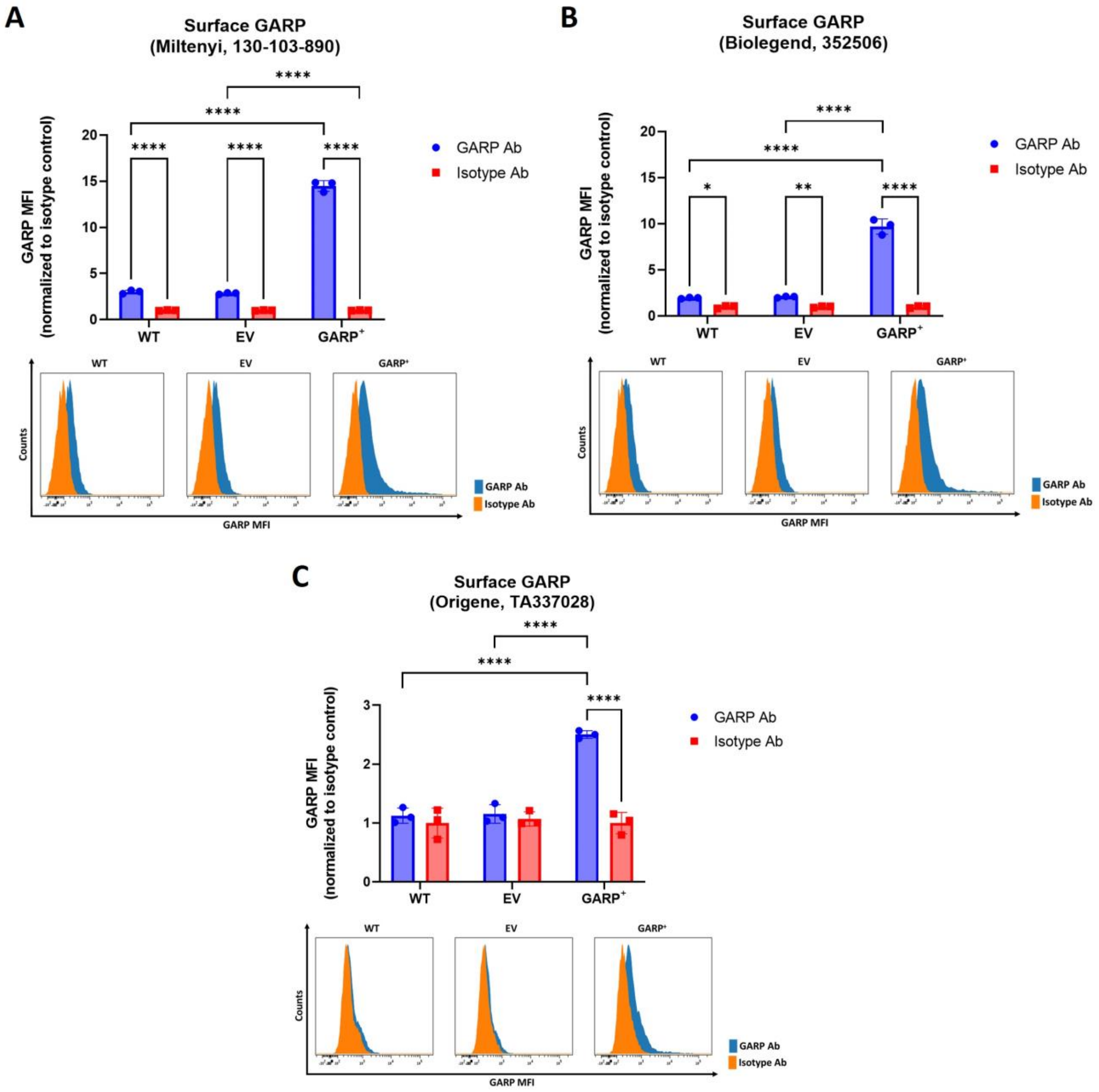

Figure S3: Specificity demonstration and validation of anti-GARP antibodies. Comparative flow cytometric analysis of surface GARP levels on wildtype (WT) and transfected (GARP overexpression (GARP$^+$), empty vector control (EV)) Mewo cells using three different human anti-GARP antibodies. The following antibodies were analyzed: Miltenyi (130-103-890) (A), Biolegend (352502) (B), Origene (TA337028) (C). Doublets, debris, and dead cells were excluded from the analysis. Graph shows the mean fluorescence intensity (MFI) normalized to the MFI of the respective isotype control, whereas histograms display one representative result (n=3, ± SD, * $p < 0.05$, ** $p < 0.01$, and **** $p < 0.0001$ determined by two-way ANOVA).

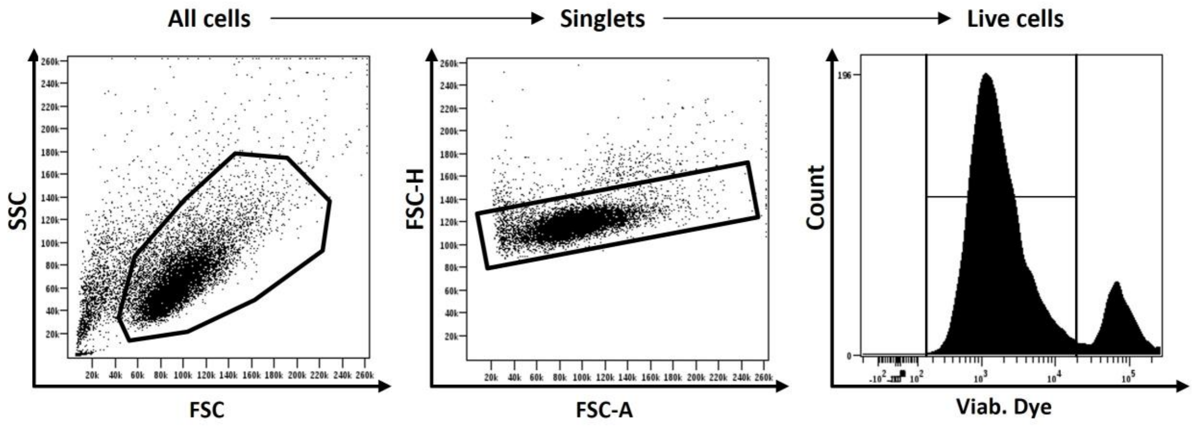

Figure S4: Flow cytometric gating strategy for GSCs. Representative flow cytometric gating strategies used for GSCs. Debris, doublets, and dead cells were excluded from analysis.

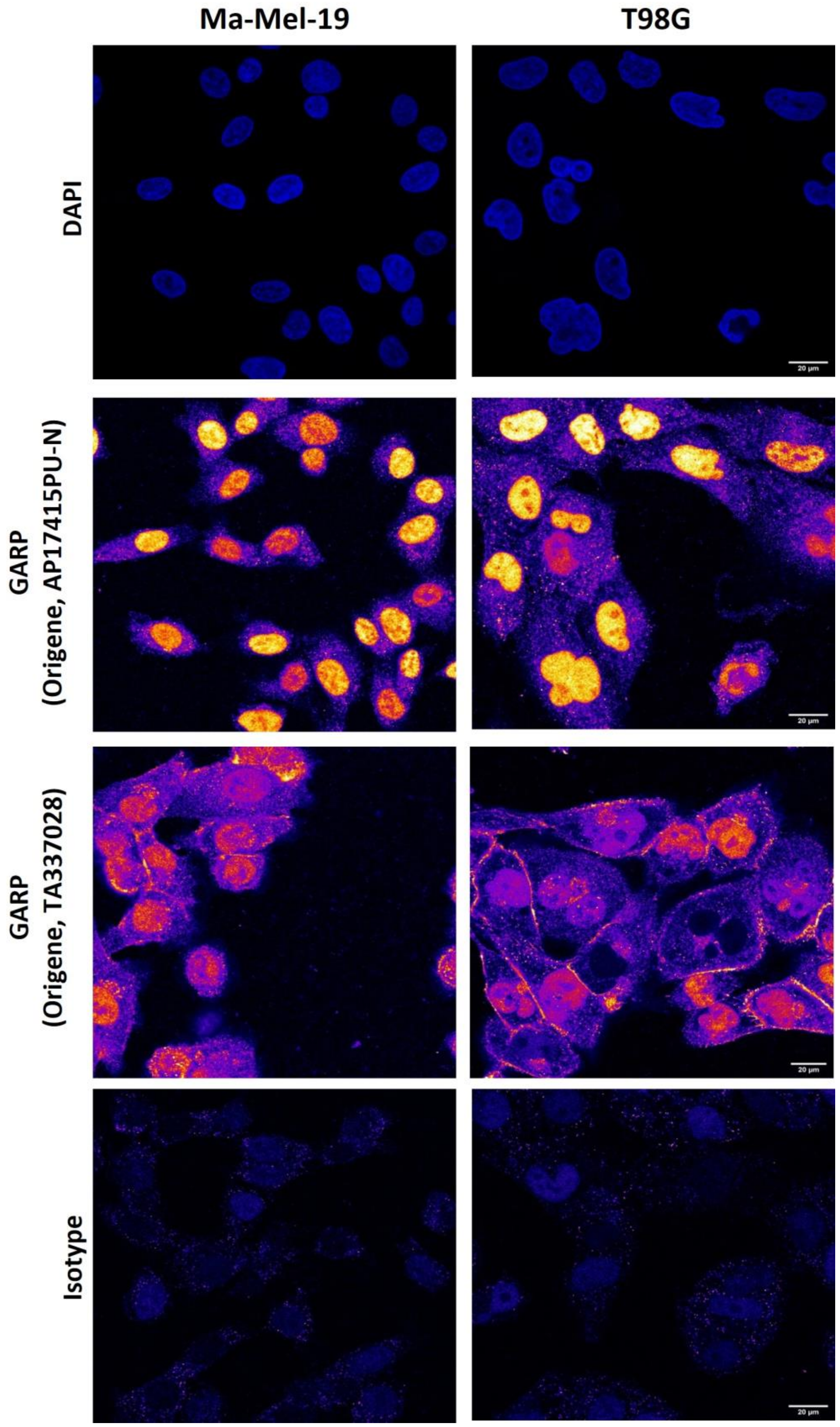

Figure S5: Anti-GARP antibody validation for confocal microscopy. Confocal images of the human GARP expressing cell lines, Ma-Mel-19 and T98G. Cells were stained for GARP using two different antibodies (Origene, AP17415PU-N; Origene, TA337028) as seen in orange. Cells were counterstained for their nuclei with Hoechst (blue). Note the intranuclear localization of GARP ($GARP^{NU+}$) detectable with both antibodies. Scale bar corresponds to 20 μm.

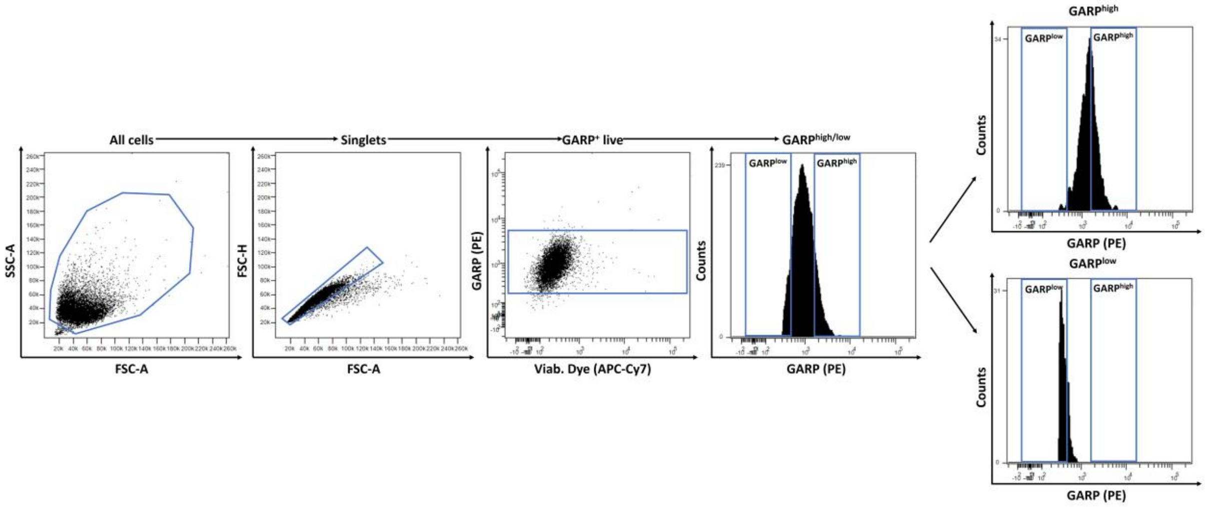

Figure S6: Flow cytometric gating strategy for GARP^{high} and GARP^{low} sorted GSCs. (A) Representative flow cytometric gating strategy used for sorting GARP^{high} and GARP^{low} GSCs. Sorted cells were re-measured via flow cytometry to confirm sorting efficacy. (B) Example GARP staining of GSCs (mean fluorescence intensity shown) compared to its respective isotype and unstained controls. Debris, doublets, and dead cells were excluded from analysis.

#1051

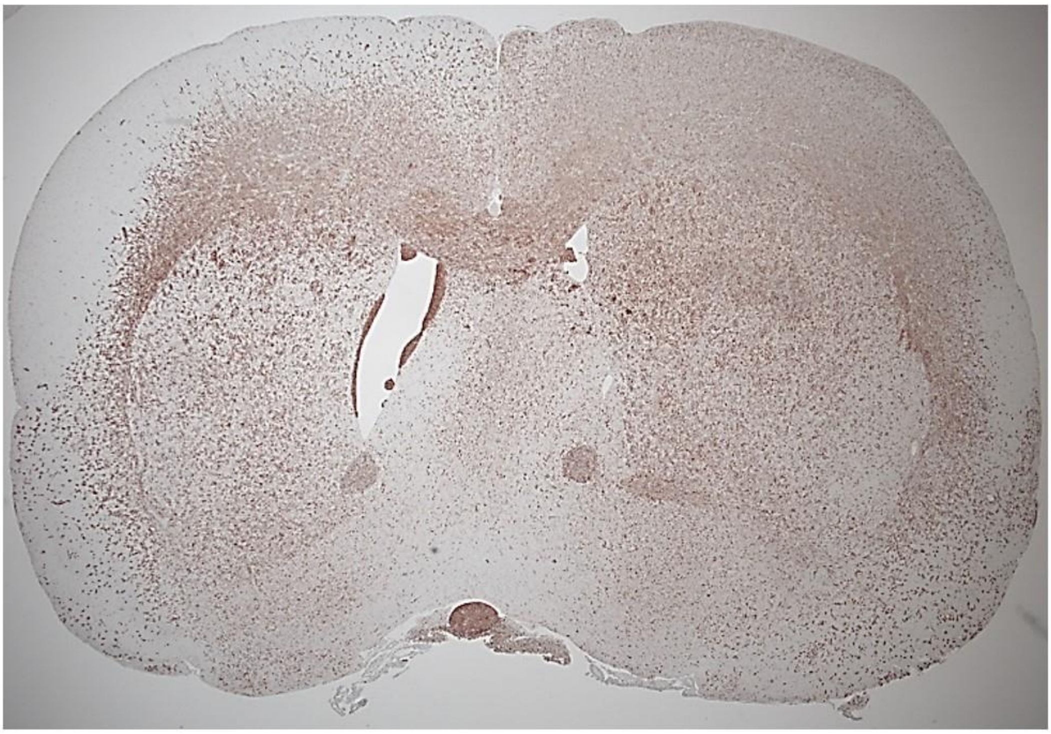

#1043

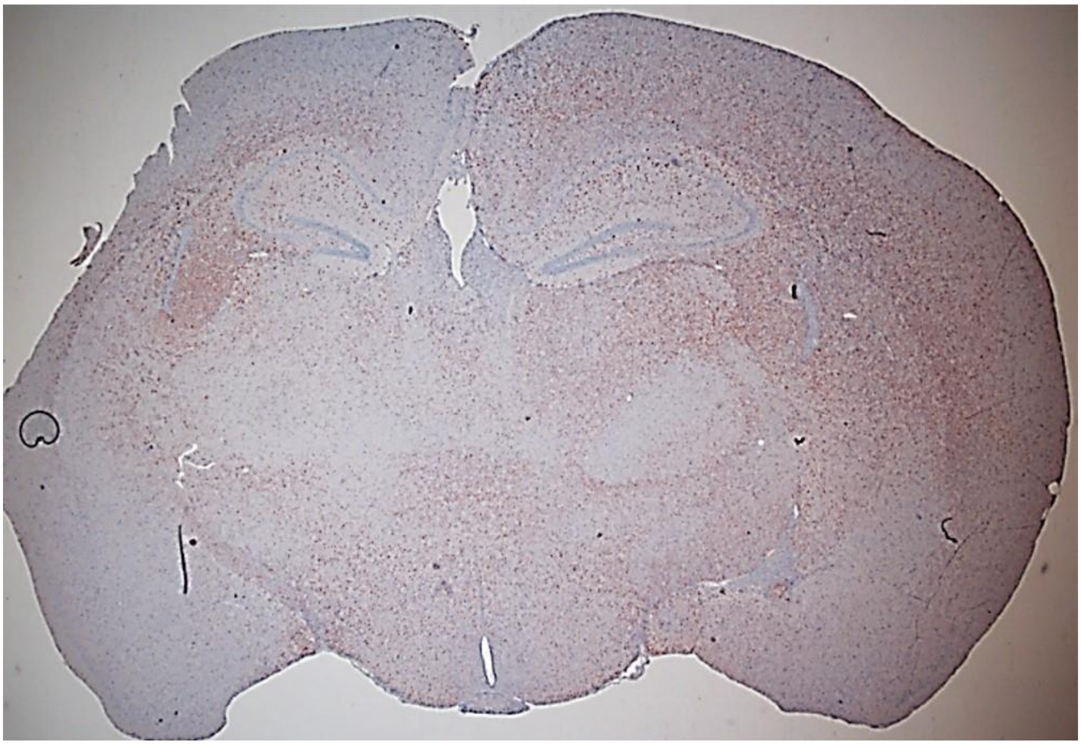

Figure S7: Invasive xenograft tumors arisen from GSC lines, #1051 and #1043. Representative images of xenograft tumors grown from human GSCs in an orthotopic mouse model for brain tumors. Immunohistochemistry stainings for human nestin (anti-human nestin antibody PA5-82905, 1:100, Life Technologies).

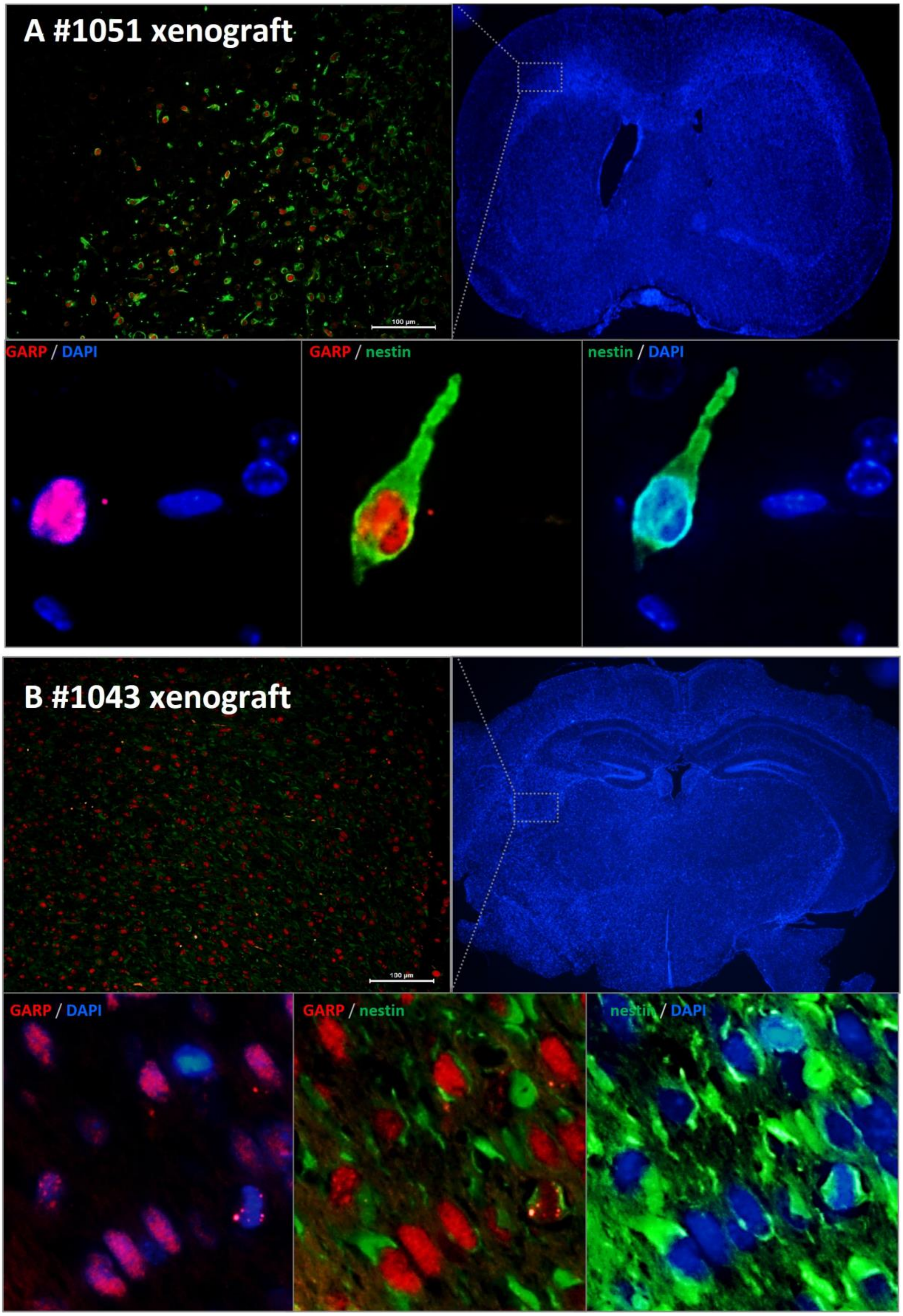

Figure S8: GARP is expressed in xenograft tumors arisen from GSC lines, #1051 and #1043. Immunofluorescence of GARP and nestin of (A) #1051 and (B) #1043 xenograft tumors. GARP seems to be exclusively expressed on GSC cells. Confocal images of GARP and nestin expressing GSCs stained for GARP and nestin. Cells were stained for their nuclei. Nuclear counterstaining with Hoechst (blue), GARP (red), and nestin (green). Scale bar corresponds to 100 µm.

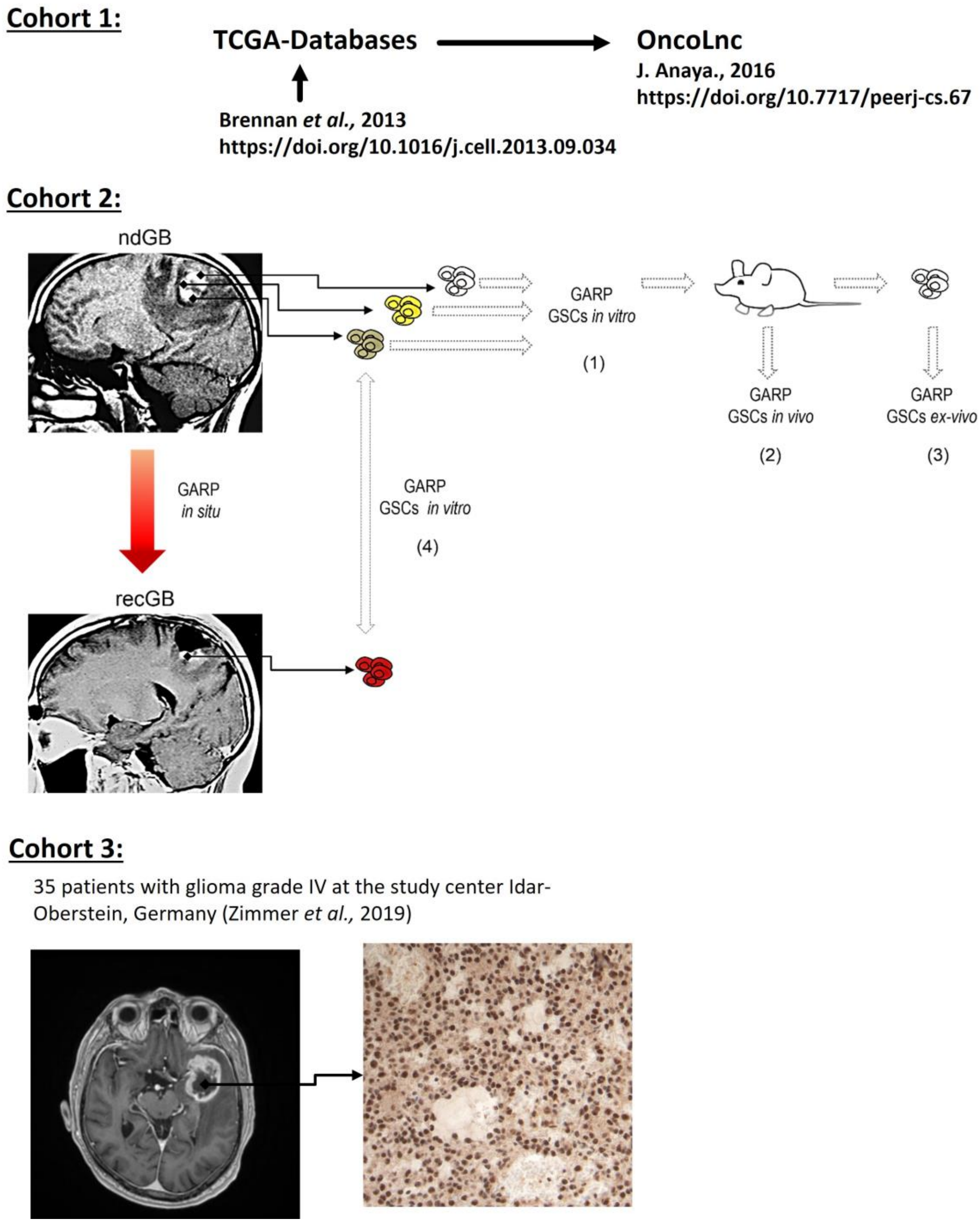

Figure S9: Study design and models used for the assessment of GARP. Cohort 1: For the analysis of GARP and CD133 expression in GB, the online tool OncoLnc was used. Based on 152 complete data sets, including complete survival data, patients were divided 50/50 into "low" or "high" groups based off their mRNA expression of GARP and CD133 and were analyzed for their survival. The results shown are in whole or part based upon data generated by the TCGA Research Network: https://www.cancer.gov/tcga and were analyzed using OncoLnc [43]. Cohort 2: „GARP in situ" corresponds to GARP assessments in tumor specimens from newly diagnosed or recurrent GBs. Investigation track (1) corresponds to *in vitro* assessments in GSCs either isogenic or heterogenic originating from ndGBs. Track (2) corresponds to *in vivo* assessments of GARP in tumor xenografts grown from orthotopically implanted GSCs. Track (3) corresponds to GARP assessments in GSCs explanted from tumor xenografts. Track (4) corresponds to tumor-matched GSCs isolated from the same patient at the ndGB or recGB stage. Furthermore, retrospective analysis of transcriptome data of 155 GB samples from 28 patients of Kim et al., 2020. ndGBs, first and second recurrent tumors were analyzed for their GARP and CD133 expression levels across tumor stages [32]. Cohort 3: A cohort of 35 patients with (WHO grade IV) glioblastoma (Zimmer et al., 2019) were analyzed for their GARP expression by immunohistochemistry and analyzed for their survival [7].

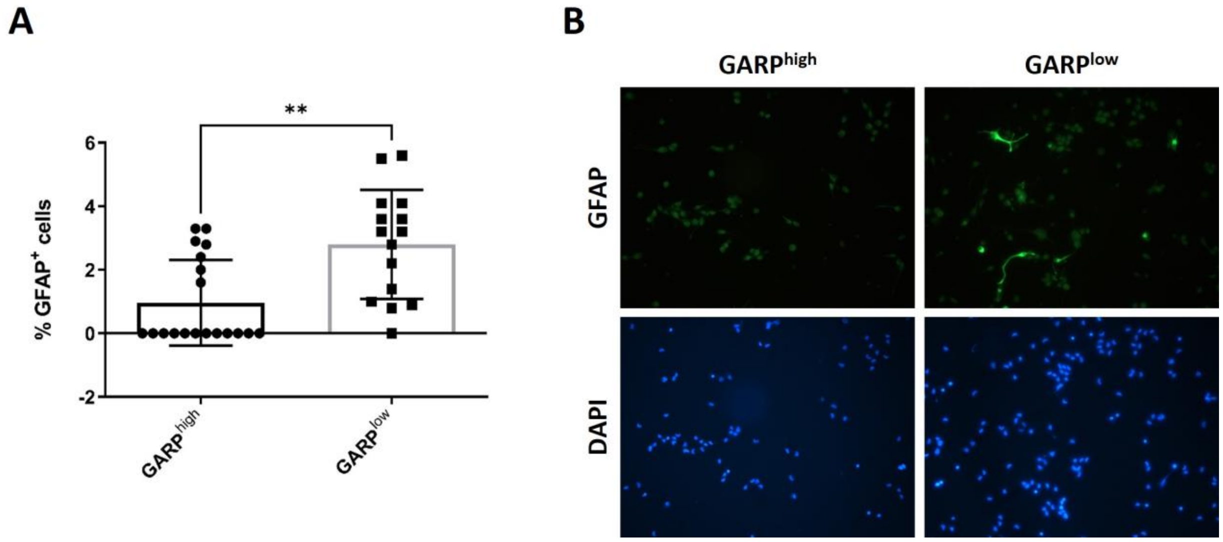

Figure S10: Frequency of GFAP+ GARPhigh and GARPlow GSCs. The GSC line, #1095, was sorted into GARPhigh and GARPlow populations. Cells were cultured in self-renewal promoting conditions (NB+bFGF/+EGF) and assessed for their frequency of glial fibrillary acidic protein (GFAP), an astrocyte differentiation associated marker, via immunofluorescence. (A) The percentage of GFAP+ cells was quantified from the total cells counted. (B) Representative images of GFAP (green) stained GARPhigh and GARPlow GSCs with paired DAPI (blue) controls. The white scale bar corresponds to 50 μm.
